# Diversity and evolution of the transposable element repertoire in arthropods with particular reference to insects

**DOI:** 10.1186/s12862-018-1324-9

**Published:** 2019-01-09

**Authors:** Malte Petersen, David Armisén, Richard A. Gibbs, Lars Hering, Abderrahman Khila, Georg Mayer, Stephen Richards, Oliver Niehuis, Bernhard Misof

**Affiliations:** 1 University of Bonn, Bonn, Germany; 2 Université de Lyon, Institut de Génomique Fonctionnelle de Lyon, CNRS UMR 5242, Ecole Normale Supérieure de Lyon, Université Claude Bernard Lyon 1, 46 allée d’Italie, Lyon, 69364 France; 3 Human Genome Sequencing Center, Department of Human and Molecular Genetics, Baylor College of Medicine, Houston, 77030 TX USA; 4 Department of Zoology, Institute of Biology, University of Kassel, Heinrich-Plett-Str. 40, Kassel, 34132 Germany; 5 Université de Lyon, Institut de Génomique Fonctionnelle de Lyon, CNRS UMR 5242, Ecole Normale Supérieure de Lyon, Université Claude Bernard Lyon 1, 46 allée d’Italie, Lyon, 69364 France; 6 Department of Zoology, Institute of Biology, University of Kassel, Heinrich-Plett-Str. 40, Kassel, 34132 Germany; 7 Human Genome Sequencing Center, Department of Human and Molecular Genetics, Baylor College of Medicine, Houston, 77030 TX USA; 8 Department of Evolutionary Biology and Ecology, Institute for Biology I (Zoology), University of Freiburg, Freiburg (Brsg.), 79104 Germany; 9 Zoological Research Museum Alexander Koenig, Center for Molecular Biodiversity Research, Adenauerallee 160, Bonn, 53113 Germany; 10 Senckenberg Gesellschaft für Naturforschung, Senckenberganlage 25, Frankfurt, 60325 Germany

## Abstract

**Background:**

Transposable elements (TEs) are a major component of metazoan genomes and are associated with a variety of mechanisms that shape genome architecture and evolution. Despite the ever-growing number of insect genomes sequenced to date, our understanding of the diversity and evolution of insect TEs remains poor.

**Results:**

Here, we present a standardized characterization and an order-level comparison of arthropod TE repertoires, encompassing 62 insect and 11 outgroup species. The insect TE repertoire contains TEs of almost every class previously described, and in some cases even TEs previously reported only from vertebrates and plants. Additionally, we identified a large fraction of unclassifiable TEs. We found high variation in TE content, ranging from less than 6% in the antarctic midge (Diptera), the honey bee and the turnip sawfly (Hymenoptera) to more than 58% in the malaria mosquito (Diptera) and the migratory locust (Orthoptera), and a possible relationship between the content and diversity of TEs and the genome size.

**Conclusion:**

While most insect orders exhibit a characteristic TE composition, we also observed intraordinal differences, e.g., in Diptera, Hymenoptera, and Hemiptera. Our findings shed light on common patterns and reveal lineage-specific differences in content and evolution of TEs in insects. We anticipate our study to provide the basis for future comparative research on the insect TE repertoire.

**Electronic supplementary material:**

The online version of this article (10.1186/s12862-018-1324-9) contains supplementary material, which is available to authorized users.

## Introduction

Repetitive elements, including transposable elements (TEs), are a major sequence component of eukaryote genomes. In vertebrate genomes, for example, the TE content varies from 6% in the pufferfish *Tetraodon nigroviridis* to more than 55% in the zebrafish *Danio rerio* [1]. More than 45% of the human genome [2] consist of TEs. In plants, TEs are even more prevalent: up to 90% of the maize (*Zea mays*) genome is covered by TEs [3]. In insects, the genomic portion of TEs ranges from as little as 1% in the antarctic midge [4] to as large as 65% in the migratory locust [5].

TEs are known as “jumping genes” and traditionally viewed as selfish parasitic nucleotide sequence elements propagating in genomes with mainly deleterious or at least neutral effects on host fitness [6, 7] (reviewed in [8]). Due to their propagation in the genome, TEs are thought to have a considerable influence on the evolution of the host’s genome architecture. By transposing into, for example, host genes or regulatory sequences, TEs can disrupt coding sequences or gene regulation, and/or provide hot spots for ectopic (non-homologous) recombination that may induce chromosomal rearrangements in the host genome such as deletions, duplications, inversions, and translocations [9]. For example, the shrinkage of the Y chromosome in the fruit fly *Drosophila melanogaster*, which consists mostly of TEs, is thought to be caused by such intrachromosomal rearrangements induced by ectopic recombination [10, 11]. As such potent agents for mutation, TEs are also responsible for cancer and genetic diseases in humans and other organisms [12–14].

Despite the potential deleterious effects of their activity on gene regulation, there is growing evidence that TEs can also be drivers of genomic innovation that confer selective advantages to the host [15, 16]. For example, it is well documented that the frequent cleavage and rearrangement of DNA strands induced by TE insertions provides a source of sequence variation to the host genome, or that by a process called molecular domestication of TEs, host genomes derive new functional genes and regulatory networks [17–19]. Furthermore, many exons have been de novo-recruited from TE insertions in coding sequences of the human genome [20]. In insects, TE insertions have played a pivotal role in the acquisition of insecticide resistance [21–23], as well as in the rewiring of a regulatory network that provides dosage compensation [24], or the evolution of climate adaptation [25, 26].

TEs are classified depending on their mode of transposition. Class I TEs, also known as retrotransposons, transpose via an RNA-mediated mechanism that can be circumscribed as “copy-and-paste”. They are further subdivided into long terminal repeat (LTR) retrotransposons and non-LTR retrotransposons. Non-LTR retrotransposons include long and short interspersed nuclear elements (LINEs and SINEs) [27, 28]. Whereas LTR retrotransposons and LINEs encode a reverse transcriptase, the non-autonomous SINEs rely on the transcriptional machinery of autonomous elements, such as LINEs, for mobility. Frequently found LTR retrotransposon families in eukaryote genomes include Ty3/Gypsy, which was originally described in *Arabidopsis thaliana* [29], Ty1/Copia [30], as well as BEL/Pao [31].

In Class II TEs, also termed DNA transposons, the transposition is DNA-based and does not require an RNA intermediate. Autonomous DNA transposons encode a transposase enzyme and move via a “cut-and-paste” mechanism. During replication, terminal inverted repeat (TIR) transposons and Crypton-type elements cleave both DNA strands [32]. Helitrons, also known as rolling-circle (RC) transposons due to their characteristic mode of transposition [33], and the self-synthesizing Maverick/Polinton elements [34] cleave a single DNA strand in the process of replication. Both Helitron and Maverick/Polinton elements occur in autonomous and non-autonomous versions [35, 36], the latter of which do not encode all proteins necessary for transposition. Helitrons are the only Class II transposons that do not cause a flanking target site duplication when they transpose. Class II also encompasses other non-autonomous DNA transposons such as miniature inverted TEs (MITEs) [37], which exploit and rely on the transposase mechanisms of autonomous DNA transposons to replicate.

Previous reports on insect genomes describe the composition of TE families in insect genomes as a mixture of insect specific TEs and TEs common to metazoa [38–40]. Overall, surprisingly little effort has been put into characterizing TE sequence families and TE compositions in insect genomes in large-scale comparative analyses encompassing multiple taxonomic orders to paint a picture of the insect TE repertoire. Dedicated comparative analyses of TE composition have been conducted on species of mosquitoes [41], of drosophilid flies [42], and of *Macrosiphini* (aphids) [43]. Despite these efforts in characterizing TEs in insect genomes, still little is known about the diversity of TEs in insect genomes, owed in part to the huge insect species diversity and to the lack of a standardized analysis that allows comparisons across taxonomic orders. While this lack of knowledge is due to the low availability of sequenced insect genomes in the past, efforts such as the i5k initiative [44] have helped to increase the number of genome sequences from previously unsampled insect taxa. With this denser sampling of insect genomic diversity available, it now seems possible to comprehensively investigate the TE diversity among major insect lineages.

Here, we present the first exhaustive analysis of the distribution of TE classes in a sample representing half of the currently classified insect (hexapod *sensu* Misof et al. [45]) orders and using standardized comparative methods implemented in recently developed software packages. Our results show similarities in TE family diversity and abundance among the investigated insect genomes, but also profound differences in TE activity even among closely related species.

## Results

### Diversity of TE content in arthropod genomes

**Fig. 1:**
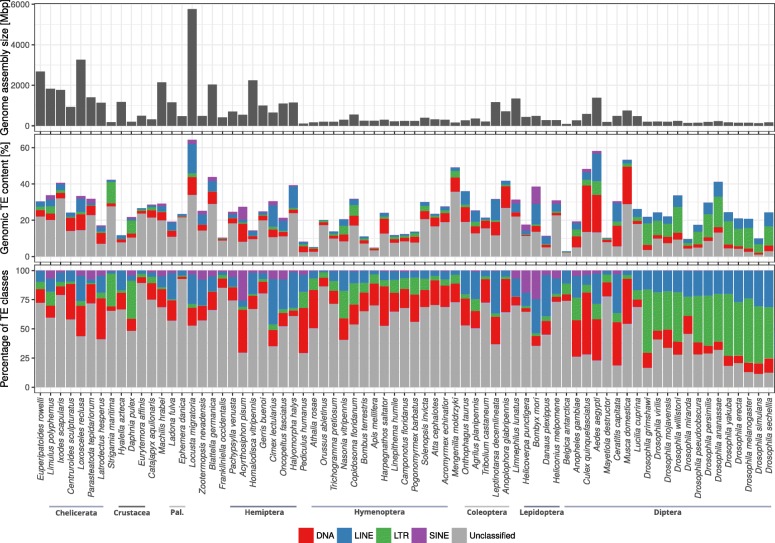
Genome assembly size, total amount and relative proportion of DNA transposons, LTR, LINE and SINE retrotransposons in arthropod genomes and a representative of Onychophora as an outgroup. Also shown is the genomic proportion of unclassified/uncharacterized repetitive elements. Pal., Palaeoptera

TE content varies greatly among the analyzed species (Fig. 1, Additional file 1: Table S1) and differs even between species belonging the same order. In the insect order Diptera, for example, the TE content varies from around 55% in the yellow fever mosquito *Aedes aegypti* to less than 1% in *Belgica antarctica*. Even among closely related *Drosophila* species, the TE content ranges from 40 % (in *D. ananassae*) to 10% (in *D. miranda* and *D. simulans*). The highest TE content (60%) was found in the large genome (6.5 Gbp) of the migratory locust *Locusta migratoria* (Orthoptera), while the smallest known insect genome, that of the antarctic midge *B. antarctica* (Diptera, 99 Mbp), was found to contain less than 1% TEs. The TE content of the majority of the genomes was spread around a median of 24.4% with a standard deviation of 12.5%.

### Relative contribution of different TE types to arthropod genome sequences

We assessed the relative contribution of the major TE groups (LTR, LINE, SINE retrotransposons, and DNA transposons) to the arthropod genome composition (Fig. 1). In most species, “unclassified” elements, which need further characterization, represent the largest fraction. They contribute up to 93% of the total TE coverage in the mayfly *Ephemera danica* or the copepod *Eurytemora affinis*. Unsurprisingly, in most investigated *Drosophila* species the unclassifiable elements comprise less than 25% and in *D. simulans* only 11% of the entire TE content, likely because the *Drosophila* genomes are well annotated and most of their content is known (in fact, many TEs were first found in representatives of *Drosophila*). Disregarding these unclassified TE sequences, LTR retrotransposons dominate the TE content in representatives of Diptera, in some cases contributing around 50% (e.g., in *D. simulans*). In Hymenoptera, on the other hand, DNA transposons are more prevalent, such as 35.25% in Jerdon’s jumping ant *Harpegnathos saltator*. LINE retrotransposons are represented with up to 39.3% in Hemiptera and Psocodea (*Acyrthosiphon pisum* and *Cimex lectularius*), with the exception of the human body louse *Pediculus humanus*, where DNA transposons contribute 44.43% of the known TE content. SINE retrotransposons were found in all insect orders, but they contributed less than 10% of the genomic TE content in any taxon in our sampling, with the exception of *Helicoverpa punctigera* (18.48%), *Bombyx mori* (26.38%), and *A. pisum* (27.11%). In some lineages, such as Hymenoptera and most dipterans, SINEs contribute less than 1% to the TE content, whereas in Hemiptera and Lepidoptera the SINE coverage ranges from 0.08% to 26.38% (Hemiptera) and 3.35 to 26.38% (Lepidoptera). Note that these numbers are likely higher and many more DNA, LTR, LINE, and SINE elements may be obscured by the large “unclassified” portion.

### Contribution of TEs to arthropod genome size

**Fig. 2:**
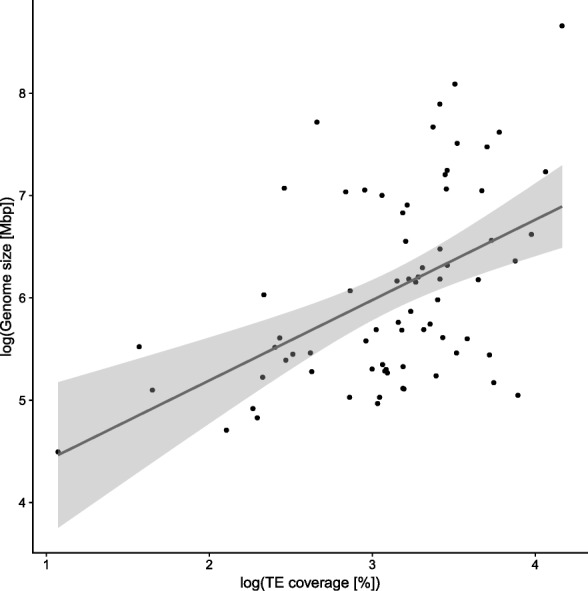
TE content in 73arthropod genomes is positively correlated to genome assembly size (Spearman rank correlation test, *ρ*=0.495, *p*⋘0.005). This correlation is also supported under phylogenetically independent contrasts [48] (Pearson product moment correlation, *ρ*=0.497, *p*=0.0001225). Dots: Individual measurements; blue line: linear regression; grey area: confidence interval

We assessed the TE content, that is, the ratio of TE versus non-TE nucleotides in the genome assembly, in 62 hexapod (insects *sensu* [45]) species as well as an outgroup of 10 non-insect arthropods and a representative of Onychophora (velvet worms). We tested whether there was a relationship between TE content and genome assembly size, and found a positive correlation (Fig. 2 and Additional file 1: Table S1). This correlation is statistically significant (Spearman’s rank sum test, *ρ*=0.495, *p*⋘0.005). Genome size is significantly smaller in holometabolous insects than in non-holometabolous insects (one-way ANOVA, *p*=0.0001). Using the ape package v. 4.1 [46] for R [47], we tested for correlation between TE content and genome size using phylogenetically independent contrasts (PIC) [48]. The test confirmed a significant positive correlation (Pearson product-moment correlation, *ρ*=0.497, *p*=0.0001, corrected for phylogeny using PIC) between TE content and genome size. Additionally, genome size is correlated with TE diversity, that is, the number of different TE superfamilies found in a genome (Spearman, *ρ*=0.712, *p*⋘0.005); this is also true under PIC (Pearson, *ρ*=0.527, *p*⋘0.005; Additional file 2: Figure S1).

### Distribution of TE superfamilies in arthropods

**Fig. 3:**
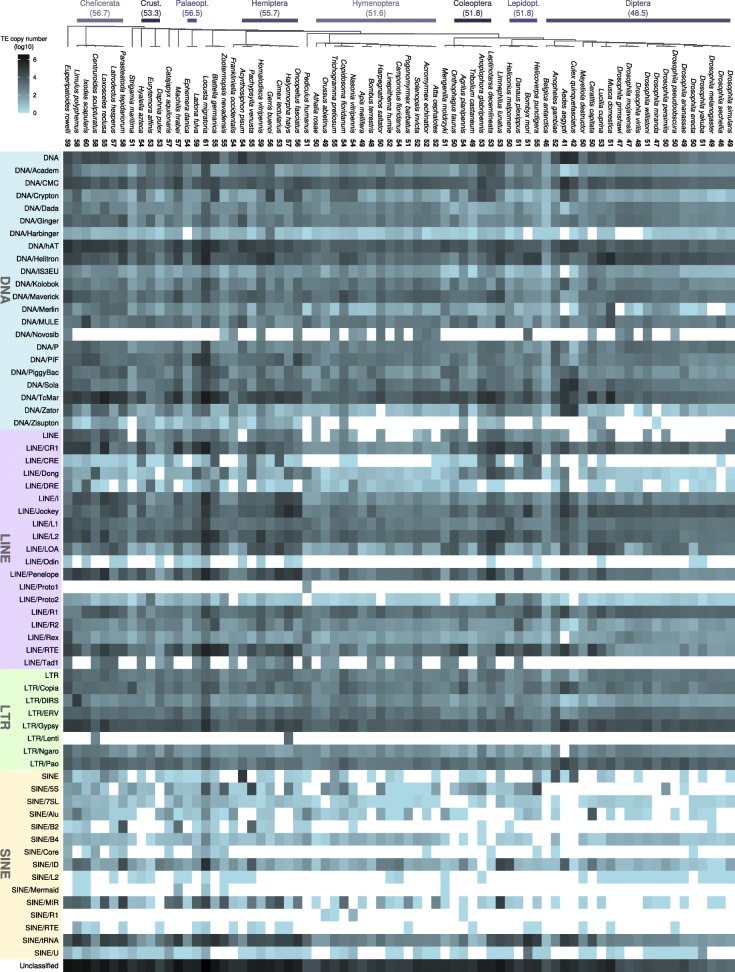
TE diversity in arthropod genomes: Many known TE superfamilies were identified in almost all insect species. Presence of TE superfamilies is shown as filled cells with the color gradient showing the TE copy number (log11). Empty cells represent absence of TE superfamilies. The numbers after each species name show the number of different TE superfamilies; numbers in parentheses below clade names denote the average number of TE superfamilies in the corresponding taxon

**Fig. 4:**
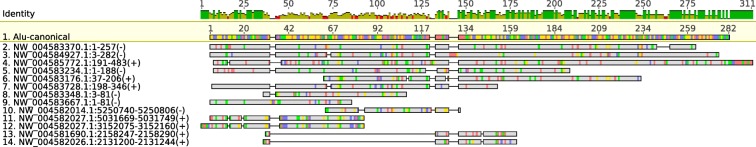
The Alu element found in *Bombyx mori*: Alignment of the canonical Alu sequence from Repbase with HMM hits in the *B. mori* genome assembly. Grey areas in the sequences are identical to the canonical Alu sequence. The sequence names follow the pattern “identifier:start-end(strand)” Image created using Geneious version 7.1 created by Biomatters. Available from https://www.geneious.com

We identified almost all known TE superfamilies in at least one insect species, and many were found to be widespread and present in all investigated species (Fig. 3, note that in this figure, TE families were summarized in superfamilies). Especially diverse and ubiquitous are DNA transposon superfamilies, which represent 22 out of 70 identified TE superfamilies. The most widespread (present in all investigated species) DNA transposons belong to the superfamilies Academ, Chapaev and other superfamilies in the CMC complex, Crypton, Dada, Ginger, hAT (Blackjack, Charlie, *etc.*), Kolobok, Maverick, Harbinger, PiggyBac, Helitron (RC), Sola, TcMar (Mariner, Tigger, *etc.*), and the P element superfamily. LINE non-LTR retrotransposons are similarly ubiquitous, though not as diverse. Among the most widespread LINEs are TEs belonging to the superfamilies CR1, Jockey, L1, L2, LOA, Penelope, R1, R2, and RTE. Of the LTR retrotransposons, the most widespread are in the superfamilies Copia, DIRS, Gypsy, Ngaro, and Pao as well as endogenous retrovirus particles (ERV). SINE elements are diverse, but show a more patchy distribution, with only the tRNA-derived superfamily present in all investigated species. We found elements belonging to the ID superfamily in almost all species except the Asian long horned beetle, *Anoplophora glabripennis*, and the B4 element absent from eight species. All other SINE superfamilies are absent in at least 13 species. Elements from the Alu superfamily were found in 48 arthropod genomes, for example in the silkworm *Bombyx mori* (Fig. 4, all Alu alignments are shown in Additional file 3).

On average, the analyzed species harbor a mean of 54.8 different TE superfamilies, with the locust *L. migratoria* exhibiting the greatest diversity (61 different TE superfamilies), followed by the tick *Ixodes scapularis* (60), the velvet worm *Euperipatoides rowelli* (59), and the dragonfly *Ladona fulva* (59). Overall, Chelicerata have the highest average TE superfamily diversity (56.7). The greatest diversity among the multi-representative hexapod orders was found in Hemiptera (55.7). The mega-diverse insect orders Diptera, Hymenoptera, and Coleoptera display a relatively low diversity of TE superfamilies (48.5, 51.8, and 51.8, respectively). The lowest diversity was found in *A. aegypti*, with only 41 TE superfamilies.

### Lineage-specific TE presence and absence in insect orders

We found lineage-specific TE diversity within most insect orders. For example, the LINE superfamily Odin is absent in all Hymenoptera studied, whereas Proto2 was found in all Hymenoptera except in the ant *H. saltator* and in all Diptera except in *C. quinquefasciatus*. Similarly, the Harbinger DNA element superfamily was found in all Lepidoptera except for the silkworm *B. mori*. Also within Palaeoptera (i.e., mayflies, damselflies, and dragonflies), the Harbinger superfamily is absent in *E. danica*, but present in all other representatives of Palaeoptera. These clade-specific absences of a TE superfamily may be the result of lineage-specific TE extinction events during the evolution of the different insect orders. Note that since a superfamily can encompass multiple different TEs, the absence of a specific superfamily can either result from independent losses of multiple TEs belonging to that superfamily, or a single loss if there only was a single TE of that superfamily in the genome.

We also found TE superfamilies represented only in a single species of an insect clade. For example, the DNA element superfamily Zisupton was found only in the wasp *Copidosoma floridanum*, but not in other Hymenoptera, and the DNA element Novosib was found only in *B. mori*, but not in other Lepidoptera. Within Coleoptera, only the Colorado potato beetle, *Leptinotarsa decemlineata* harbors the LINE superfamily Odin. Likewise, we found the Odin superfamily among Lepidoptera only in the noctuid *Helicoverpa punctigera*. We found the LINE superfamily Proto1 only in *Pediculus humanus* and in no other species. These examples of clade or lineage specific occurrence of TEs, which are absent from other species of the same order (or the entire taxon sampling), could be the result of a horizontal transfer from food species or a bacterial/viral infection.

### Lineage-specific TE activity during arthropod evolution

**Fig. 5:**
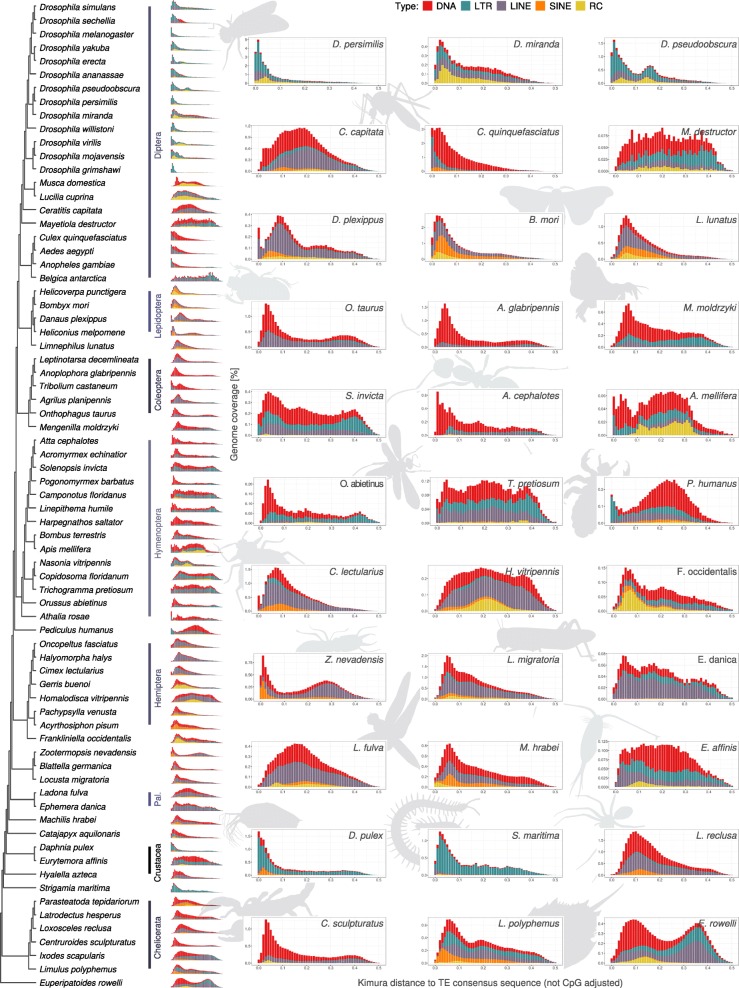
Cladogram with repeat landscape plots. The larger plots are selected representatives. The further to the left a peak in the distribution is, the younger the corresponding TE fraction generally is (low TE intra-family sequence divergence). In most orders, the TE divergence distribution is similar, such as in Diptera or Hymenoptera. The large fraction of unclassified elements was omitted for these plots. Pal., Palaeoptera

We further analyzed sequence divergence measured by Kimura distance within each species-specific TE content (Fig. 5; note that for these plots, we omitted the large fraction of unclassified elements). Within Diptera, the most striking feature is that almost all investigated drosophilids show a large spike of LTR retroelement proliferation between Kimura distance 0 and around 0.08. This spike is only absent in *D. miranda*, but bi-modal in *D. pseudoobscura*, with a second peak around Kimura distance 0.15. This second peak, however, does not coincide with the age of inversion breakpoints on the third chromosome of *D. pseudoobscura*, which are only a million years old and have been associated with TE activity [49]. A bi-modal distribution was not observed in any other fly species. On the contrary, all mosquito species exhibit a large proportion of DNA transposons which show a divergence between Kimura distance 0.02 and around 0.3. This divergence is also present in the calyptrate flies *Musca domestica*, *Ceratitis capitata*, and *Lucilia cuprina*, but absent in all acalyptrate flies, including representatives of the *Drosophila* family. Likely, the LTR proliferation in drosophilids as well as the DNA transposon expansion in mosquitos and other flies was the result of a lineage-specific invasion and subsequent propagation into the different dipteran genomes.

In the calyptrate flies, Helitron elements are highly abundant, representing 28% of the genome in the house fly *M. domestica* and 7% in the blow fly *Lucilia cuprina*. These rolling circle elements are not as abundant in acalyptrate flies, except for the drosophilids *D. mojavensis*, *D. virilis*, *D. miranda*, and *D. pseudoobscura* (again with a bi-modal distribution). In the barley midge, *Mayetiola destructor*, DNA transposons occur across almost all Kimura distances between 0.02 and 0.45. The same holds true for LTR retrotransposons, although these show an increased expansion in the older age categories at Kimura distances between 0.37 and 0.44. LINEs and SINEs as well as Helitron elements show little occurrence in Diptera. In *B. antarctica*, LINE elements are the most prominent and exhibit a distribution across all Kimura distances up to 0.4. This may be a result of the overall low TE concentration in the small *B. antarctica* genome (less than 1%) that introduces stochastic noise.

In Lepidoptera, we found a relatively recent SINE expansion event around Kimura distance 0.03 to 0.05. In fact, Lepidoptera and Trichoptera are the only holometabolous insect orders with a substantial SINE portion of up to 9% in the silk worm *B. mori* (mean: 3.8%). We observed that in the postman butterfly, *Heliconius melpomene*, the SINE fraction also appears with a divergence between Kimura distances 0.1 to around 0.31. Additionally, we found high LINE content in the monarch butterfly *Danaus plexippus* with a divergence ranging from Kimura distances 0 to 0.47 and a substantial fraction around Kimura distance 0.09.

In all Coleoptera species, we found substantial LINE and DNA content with a divergence around Kimura distance 0.1. In the beetle species *Onthophagus taurus*, *Agrilus planipennis*, and *L. decemlineata*, this fraction consists mostly of LINE copies, while in *T. castaneum* and *A. glabripennis* DNA elements make up the major fraction. In all Coleoptera species, the amount of SINEs and Helitrons is small (cf. Fig. 1). Interestingly, *Mengenilla moldrzyki*, a representative of Strepsiptera, which was previously determined to be the sister group of Coleoptera [50], shows more similarity in TE divergence distribution to Hymenoptera than to Coleoptera, with a large fraction of DNA elements covering Kimura distances 0.05 to around 0.3 and relatively small contributions from LINEs.

In apocritan Hymenoptera (i.e., those with a wasp waist), the DNA element divergence distribution exhibits a peak around Kimura distance 0.01 to 0.05. In fact, the TE divergence distribution looks very similar among the ants and differs mostly in absolute coverage, except in *Camponotus floridanus*, which shows no such distinct peak. Instead, in *C. floridanus*, we found DNA elements and LTR elements with a relatively homogeneous coverage distribution between Kimura distances 0.03 and 0.4. *C. floridanus* is also the only hymenopteran species with a noticeable SINE proportion; this fraction’s peak divergence is around Kimura distance 0.05. The relatively TE-poor genome of the honey bee, *Apis mellifera* contains a large fraction of Helitron elements with a Kimura distance between 0.1 and 0.35, as does *Nasonia vitripennis* with peak coverage around Kimura distance 0.15. These species-specific Helitron appearances are likely the result of an infection from a parasite or virus, as has been demonstrated in Lepidoptera [51]. In the (non-apocritan) parasitic wood wasp, *O. abietinus*, the divergence distribution is similar to that in ants, with a dominant DNA transposon coverage around Kimura distance 0.05. The turnip sawfly, *A. rosae* has a large, zero-divergence fraction of DNA elements, LINEs and LTR retrotransposons followed by a bi-modal divergence distribution of DNA elements.

When examining Hemiptera, Thysanoptera, and Psocodea, the DNA element fraction with high divergence (peak Kimura distance 0.25) sets the psocodean *P. humanus* apart from Hemiptera and Thysanoptera. Additionally, *P. humanus* exhibits a large peak of LTR element coverage with a low divergence (Kimura distance 0). In Hemiptera and Thysanoptera, we found DNA elements with a high coverage around Kimura distance 0.05 instead of around 0.3, like in *P. humanus*, or only in miniscule amounts, such as in *Halyomorpha halys*. Interestingly, the three bug species *H. halys*, *Oncopeltus fasciatus*, and *Cimex lectularius* show a strikingly similar TE divergence distribution which differs from that in other species of Hemiptera. In these species, the TE landscape is characterized by a wide-ranging distribution of LINE divergence with peak coverage around Kimura distance 0.07. Further, they exhibit a shallow, but consistent proportion of SINE coverage with a divergence distribution between Kimura distance 0 and around 0.3. The other species of Hemiptera and Thysanoptera show no clear pattern of similarity. In the flower thrips *Frankliniella occidentalis* (Thysanoptera) as well as in the water strider *Gerris buenoi* and the cicadellid *Homalodisca vitripennis*, (Hemiptera), the Helitron elements show a distinct coverage between Kimura distances 0 and 0.3, with peak coverage at around 0.05 to 0.1 (*F. occidentalis*, *G. buenoi*) and 0.2 (*H. vitripennis*). In both *F. occidentalis* and *G. buenoi*, the divergence distribution is slightly bi-modal. In *H. vitripennis*, LINEs and DNA elements exhibit a divergence distribution with high coverage at Kimura distances 0.02 to around 0.45. SINEs and LTR element coverage is only slightly visible. This is in stark contrast to the findings in the pea aphid *Acyrthosiphon pisum*, where SINEs make up the majority of the TE content and exhibit a broad spectrum of Kimura distances from 0 to 0.3, with peak coverage at around Kimura distance 0.05. Additionally, we found DNA elements in a similar distribution, but showing no clear peak. Instead, LINEs and LTR elements are distinctly absent from the *A. pisum* genome, possibly as a result of a lineage-specific extinction event.

The TE landscape in Polyneoptera is dominated by LINEs, which in the cockroach *Blattella germanica* have a peak coverage at around Kimura distance 0.04. In the termite *Zootermopsis nevadensis*, the peak LINE coverage is between Kimura distances 0.2 and 0.4. In the locust *L. migratoria*, LINE coverage shows a broad divergence distribution. Low-divergence LINEs show peak coverage at around Kimura distance 0.05. All three Polyneoptera species have a small, but consistent fraction of low-divergence SINE coverage with peak coverage between Kimura distances 0 to 0.05 as well as a broad, but shallow distribution of DNA element divergence.

LINEs also dominate the TE landscape in Paleoptera. The mayfly *E. danica* additionally exhibits a population of LTR elements with medium divergence in the genome. In the dragonfly *L. fulva*, we found DNA elements of similar coverage and divergence as the LTR elements. Both TE types have almost no low-divergence elements in *L. fulva*. In the early divergent apterygote hexapod orders Diplura (represented by the species *Catajapyx aquilonaris*) and Archaeognatha (*Machilis hrabei*), DNA elements are abundant with a broad divergence spectrum and low-divergence peak coverage. Additionally, we found other TE types with high coverage in low divergence regions in the genome of *C. aquilonaris* as well as SINE peak coverage at slightly higher divergence in *M. hrabei*.

The non-insect outgroup species also exhibit a highly heterogeneous TE copy divergence spectrum. In all species, we found high coverage of varying TE types with low divergence. All chelicerate genomes contain mostly DNA transposons, with LINEs and SINEs contributing a fraction in the spider *Parasteatoda tepidariorum* and the tick *I. scapularis*. The only available myriapod genome, that of the centipede *Strigamia maritima*, is dominated by LTR elements with high coverage in a low-divergence spectrum, but also LTR elements that exhibit a higher Kimura distance. We found the same in the crustacean *Daphnia pulex*, but the TE divergence distribution in the other crustacean species was different and consisted of more DNA transposons in the copepod *E. affinis*, or LINEs in the amphipod *Hyalella azteca*.

## Discussion

We used species-specific TE libraries to assess the genomic retrotransposable and transposable element content in sequenced and assembled genomes of arthropod species, including most extant insect orders.

### TE content contributes to genome size in arthropods

TEs and other types of DNA repeats are an omnipresent part of metazoan, plant, as well as fungal genomes and are found in variable proportions in sequenced genomes of different species. In vertebrates and plants, studies have shown that TE content is a predictor for genome size [1, 52]. For insects, this has also been reported in clade-specific studies such as those on mosquitoes [41] and *Drosophila* fruit flies [42]. These observations lend further support to the hypothesis that genome size is also correlated with TE content in insects on a pan-ordinal scale.

Our analysis shows that both genome size and TE content are highly variable among the investigated insect genomes, even in comparative contexts with low variation in genome size. While non-holometabolous hexapods have a significantly smaller genome than holometabolous insects, the TE content is not significantly different. Still, we found that TE content contributes significantly to genome size in hexapods as a whole. These results are in line with prior studies on insects with a more limited taxon sampling reporting a clade-specific correlation between TE content and genome size [42, 53–57], and expand that finding to larger taxon sampling covering most major insect orders. These findings further support the hypothesis that TEs are a major factor in the dynamics of genome size evolution in Eukaryotes. While differential TE activity apparently contributes to genome size variation [58–60], whole genome duplications, such as suggested by integer-sized genome size variations in some representatives of Hymenoptera [61], segmental duplications, deletions, and other repeat proliferation [62] could contribute as well. This variety of influencing factors potentially explains the range of dispersion in the correlation.

The high range of dispersion in the correlation of TE content and genome size is most likely also amplified by heterogeneous underestimates of the genomic TE coverage. Most of the genomes were sequenced and assembled using different methods, and with insufficient sequencing depth and/or older assembly methods; the data are therefore almost certainly incomplete with respect to repeat-rich regions. Assembly errors and artifacts also add a possible error margin, as assemblers cannot reconstruct repeat regions that are longer than the insert size accurately from short reads [63–66] and most available genomes were sequenced using short read technology only. Additionally, RepeatMasker is known to underestimate the genomic repeat content [2]. By combining RepeatModeler to infer the species-specific repeat libraries and RepeatMasker to annotate the species-specific repeat libraries in the genome assemblies, our methods are purposefully conservative and may have missed some TE types, or ancient and highly divergent copies.

This underestimation of the TE content notwithstanding, we found many TE families that were previously thought to be restricted to, for example, mammals, such as the SINE family Alu [67] and the LINE family L1 [68], or to fungi, such as Tad1 [69]. Essentially, most known superfamilies were found in the investigated insect genomes (*cf*. Fig. 3) and additionally, we identified highly abundant unclassifiable TEs in all insect species. These observations suggest that the insect mobilome (the entirety of mobile DNA elements) is more diverse than the well characterized vertebrate mobilome [1] and requires more exhaustive characterization. We were able to reach these conclusions by relying on two essential non-standard analyses. First, our annotation strategy of de novo repeat library construction and classification according to the RepBase database was more specific to each genome than the default RepeatMasker analysis using only the RepBase reference library. The latter approach is usually done when releasing a new genome assembly to the public. The second difference between our approach and the conventional application of the RepBase library was that we used the entire Metazoa-specific section of RepBase instead of restricting our search to Insecta. This broader scope allowed us to annotate TEs that were previously unknown from insects, and that would otherwise have been overlooked. Additionally, by removing results that matched non-TE sequences in the NCBI database, our annotation becomes more robust against false positives. The enormous previously overlooked diversity of TEs in insects does not seem to be surprising given the geological age and species richness of this clade. Insects originated more than 450 million years ago [45] and represent over 80% of the described metazoan species [70]. Further investigations will also show whether there is a connection between TE diversity or abundance and clade-specific genetic and genomic traits, such as the sex determination system (e.g., butterflies have Z and W chromosomes instead of X and Y [71]) or the composition of telomeres, which have been shown in *D. melanogaster* to exhibit a high density of TEs [72], whereas telomeres in other insects consist mostly of simple repeats. It remains to be analyzed in detail, however, whether insect TE diversity evolved independently within insects or is the result of multiple TE introgression into insect genomes.

Our results show that virtually all known TE classes are present in all investigated insect genomes. However, a large part of the TEs we identified remains unclassifiable despite the diversity of metazoan TEs in the reference library RepBase. This abundance of unclassifiable TEs suggests that the insect TE repertoire requires more exhaustive characterization and that our understanding of the insect mobilome is far from complete.

It has been hypothesized that population-level processes might contribute to TE content differences and genome size variation in vertebrates [73]. In insects, it has been shown that TE activity also varies on the population level, for example in the genomes of *Drosophila* spp. [74–76] or in the genome of the British peppered moth *Biston betularia*, in which a tandemly repeated TE confers an adaptive advantage in response to short-term environmental changes [77]. The TE activity within populations is expected to leave footprints in the nucleotide sequence diversity of TEs in the genome as recent bursts of TEs should be detectable by a large number of TE sequences with low sequence divergence.

To explain TE proliferation dynamics, two different models of TE activity have been proposed: the equilibrium model and the burst model. In the equilibrium model, TE proliferation and elimination rates are more or less constant and cancel each other out at a level that is different for each genome [78]. In this model, differential TE elimination rate contributes to genome size variation when TE activity is constant. This model predicts that in species with a slow rate of DNA loss, genome size tends to increase [79, 80]. In the burst model, TEs do not proliferate at a constant rate, but rather in high copy rate bursts following a period of inactivity [76]. These bursts can be TE family specific. Our analysis of TE landscape diversity (see below), supports the burst hypothesis. In almost every species we analyzed, there is a high proportion of abundant TE sequences with low sequence divergence and the most abundant TEs are different even among closely related species. It was hypothesized that TE bursts enabled by periods of reduced efficiency in counteracting host defense mechanisms such as TE silencing [81, 82] have resulted in differential TE contribution to genome size.

### TE landscape diversity in arthropods

In vertebrates, it is possible to trace lineage-specific contributions of different TE types [1]. In insects, however, the TE composition shows a statistically significant correlation to genome size, but a high range of dispersion. Instead, we can show that major differences both in TE abundance and diversity exist between species of the same lineage (Fig. 3). Using the Kimura nucleotide sequence distance, we observe distinct variation, but also similarities, in TE composition and activity between insect orders and among species of the same order. The number of recently active elements can be highly variable, such as LTR retrotransposons in fruit flies or DNA transposons in ants (Fig. 5). On the other hand, the shape of the TE coverage distributions can be fairly similar among species of the same order; this is particularly visible in Hymenoptera and Diptera. These findings suggest lineage-specific similarities in TE elimination mechanisms; possibly shared efficacies in the piRNA pathway that silences TEs during transcription in metazoans (e.g., in *Drosophila* [83, 84], *B. mori* [85], *Caenorhabditis elegans* [86], and mouse [87]. Another possible explanation would be recent horizontal transfers from, for example, parasite to host species (see below).

### Can we infer an ancestral arthropod mobilome in the face of massive horizontal TE transfer?

In a purely vertical mode of TE transmission, the genome of the last common ancestor (LCA) of insects — or arthropods — can be assumed to possess a superset of the TE superfamilies present in extant insect species. As many TE families appear to have been lost due to lineage-specific TE extinction events, the ancestral TE repertoire may have been even more extensive compared with the TE repertoire of extant species and might have included almost all known metazoan TE superfamilies such as the CMC complex, Ginger, Helitron, Mavericks, Jockey, L1, Penelope, R1, DIRS, Ngaro, and Pao. Many SINEs found in extant insects were most likely part of the ancestral mobilome as well, for example Alu, which was previously thought to be restricted to primates [88], and MIR.

The mobilome in extant species, however, appears to be the product of both vertical and horizontal transmission. In contrast to a vertical mode of transmission, horizontal gene transfers, common phenomenona among prokaryotes (and making a prokaryote species phylogeny nigh meaningless) and widely occurring in plants, are rather rare in vertebrates [89, 90], but have been described in Lepidoptera [91] and other insects [92]. Recently, a study uncovered large-scale horizontal transfer of TEs (horizontal transposon transfer, HTT) among insects [93] and makes this mechanism even more likely to be the source of inter-lineage similarities in insect genomic TE composition. In the presence of massive HTT, the ancestral mobilome might be impossible to infer because the effects of HTT overshadow the result of vertical TE transfer. It remains to be analyzed in detail whether the high diversity of the insect mobilomes can be better explained by massive HTT events.

## Conclusions

The present study provides an overview of the diversity and evolution of TEs in the genomes of major lineages of extant insects. The results show that there is large intra- and inter-lineage variation in both TE content and composition. This, and the highly variable age distribution of individual TE superfamilies, indicate a lineage-specific burst-like mode of TE proliferation in insect genomes. In addition to the complex composition patterns that can differ even among species of the same genus, there is a large fraction of TEs that remain unclassified, but often make up the major part of the genomic TE content, indicating that the insect mobilome is far from completely characterized. This study provides a solid baseline for future comparative genomics research. The functional implications of lineage-specific TE activity for the evolution of genome architecture will be the focus of future investigations.

## Materials and methods

### Genomic data sets

We downloaded genome assemblies of 42 arthropod species from NCBI GenBank at ftp.ncbi.nlm.nih.gov/genomes (last accessed 2014-11-26; Additional file 4: Table S2) as well as the genome assemblies of 31 additional species from the i5k FTP server at ftp://ftp.hgsc.bcm.edu:/I5K-pilot/ (last accessed 2016-07-08; Additional file 4: Table S2). Our taxon sampling includes 21 dipterans, four lepidopterans, one trichopteran, five coleopterans, one strepsipteran, 14 hymenopterans, one psocodean, six hemipterans, one thysanopteran, one blattodean, one isopteran, one orthopteran, one ephemeropteran, one odonate, one archaeognathan, and one dipluran. As outgroups we included three crustaceans, one myriapod, six chelicerates, and one onychophoran.

### Construction of species-specific repeat libraries and TE annotation in the genomes

We compiled species-specific TE libraries using automated annotation methods. RepeatModeler Open-1.0.8 [94] was employed to cluster repetitive *k*-mers in the assembled genomes and infer consensus sequences. These consensus sequences were classified using a reference-based similarity search in RepBase Update 20140131 [95]. The entries in the resulting repeat libraries were then searched for using nucleotide BLAST in the NCBI nr database (downloaded 2016-03-17 from ftp://ftp.hgsc.bcm.edu:/I5K-pilot/) to verify that the included consensus sequences are indeed TEs and not annotation artifacts. Repeat sequences that were annotated as “unknown” and that resulted in a BLAST hit for known TE proteins such as reverse transcriptase, transposase, integrase, or known TE domains such as gag/pol/env, were kept and considered unknown TE nucleotide sequences; but all other “unknown” sequences were not considered TE sequences and therefore removed. The filter patterns are included in the data package available at the Dryad repository (see the “Availability of data and materials” section). The filtered repeat library was combined with the Metazoa-specific section of RepBase version 20140131 and subsequently used with RepeatMasker 4.0.5 [94] to annotate TEs in the genome assemblies.

### Validation of Alu presence

To exemplarily validate our annotation, we selected the SINE Alu, which was previously only identified in primates [67]. We retrieved a Hidden Markov model (HMM) profile for the AluJo subfamily from the repeat database Dfam [96] and used the HMM to search for Alu copies in the genome assemblies. We extracted the hit nucleotide subsequences from the assemblies and inferred a multiple nucleotide sequence alignment with the canonical Alu nucleotide sequence from Repbase [95].

### Genomic TE coverage and correlation with genome size

We used the tool “one code to find them all” [97] on the RepeatMasker output tables to calculate the genomic proportion of annotated TEs. “One code to find them all” is able to merge entries belonging to fragmented TE copies to produce a more accurate estimate of the genomic TE content and especially the copy numbers. To test for a relationship between genome assembly size and TE content, we applied a linear regression model and tested for correlation using the Spearman rank sum method. To see whether the genomes of holometabolous insects are different than the genomes of hemimetabolous insects in TE content, we tested for an effect of the taxa using their mode of metamorphosis as a three-class factor: Holometabola (all holometabolous insect species), non-Eumetabola (all non-holometabolous hexapod species, with the exception of Hemiptera, Thysanoptera, and Psocodea; [99]), and Acercaria (Hemiptera, Thysanoptera, and Psocodea). We also tested for a potential phylogenetic effect on the correlation between genome size and TE content with the phylogenetic independent contrasts (PIC) method proposed by Felsenstein [48] using the ape package [46] within R [47]

### Kimura distance-based TE age distribution

We used intra-family TE nucleotide sequence divergence as a proxy for intra-family TE age distributions. Sequence divergence was calculated as intra-family Kimura distances (rates of transitions and transversions) using the specialized helper scripts from the RepeatMasker 4.0.5 package. The tools compute the Kimura distance between each annotated TE copy and the consensus sequence of the respective TE family, and provide the data in tabular format for processing. When plotted (Fig. 5), a peak in the distribution shows the genomic coverage of the TE copies with that specific Kimura distance to the repeat family consensus. Thus, a large peak with high Kimura distance would indicate a group of TE copies with high sequence divergence due to genetic drift or other processes. The respective TE copies are likely older than copies associated with a peak at low Kimura distance. We used the Kimura distances without correction for CpG pairs since TE DNA methylation is clearly absent in holometabolous insects and insufficiently described in hemimetabolous insects [98]. All TE age distribution landscapes were inferred from the data obtained by annotating the genomes with de novo-generated species-specific repeat libraries.

## Additional files

Additional file 1 Statistics on the TE content of arthropod genomes. This tab-separated table lists the genome assembly size as well as the genome coverage of DNA, LINE, LTR, SINE, and Unknown transposons. (TXT 8 kB)

Additional file 2 This plot shows that the number of TE superfamilies is correlated to the genome assembly size. (PDF 7 kB)

Additional file 3 Alu alignments. These plots illustrate that copies of the SINE Alu are present in 56 of the genomes under study. Grey sections in the alignments are positions identical to the canonical Alu sequence at the top. (PDF 4480 kb)

Additional file 4 Genomic datasets. This tab-separated table contains the download URLs for the genome assemblies used in this study. (TXT 10 kB)

## Abbreviations

ANOVA: Analysis of variance
BLAST: Basic local alignment search tool
ERV: Endogenous retrovirus particle
HMM: Hidden Markov model
LCA: Last common ancestor
LINE: Long interspersed nuclear element
LTR: Long terminal repeat
MITE: Miniature inverted transposable element
NCBI: National Center for Biotechnology information
PIC: Phylogenetic independent contrasts
SINE: Short interspersed nuclear element
TE: Transposable element
