# Correction to: Diversity and evolution of the transposable element repertoire in arthropods with particular reference to insects

**DOI:** 10.1186/s12862-021-01778-w

**Published:** 2021-07-16

**Authors:** Malte Petersen, David Armisén, Richard A. Gibbs, Lars Hering, Abderrahman Khila, Georg Mayer, Stephen Richards, Oliver Niehuis, Bernhard Misof

**Affiliations:** 1 University of Bonn, Bonn, Germany; 2 Université de Lyon, Institut de Génomique Fonctionnelle de Lyon, CNRS UMR 5242, Ecole Normale Supérieure de Lyon, Université Claude Bernard Lyon 1, 46 allée d’Italie, 69364 Lyon, France; 3 Human Genome Sequencing Center, Department of Human and Molecular Genetics, Baylor College of Medicine, Houston, 77030 TX USA; 4 Department of Zoology, Institute of Biology, University of Kassel, Heinrich-Plett-Str. 40, 34132 Kassel, Germany; 5 Université de Lyon, Institut de Génomique Fonctionnelle de Lyon, CNRS UMR 5242, Ecole Normale Supérieure de Lyon, Université Claude Bernard Lyon 1, 46 allée d’Italie, 69364 Lyon, France; 6 Department of Zoology, Institute of Biology, University of Kassel, Heinrich-Plett-Str. 40, 34132 Kassel, Germany; 7 Human Genome Sequencing Center, Department of Human and Molecular Genetics, Baylor College of Medicine, Houston, TX 77030 USA; 8 Department of Evolutionary Biology and Ecology, Institute for Biology I (Zoology), University of Freiburg, 79104 Freiburg (Brsg.), Germany; 9 Zoological Research Museum Alexander Koenig, Center for Molecular Biodiversity Research, Adenauerallee 160, 53113 Bonn, Germany; 10 Senckenberg Gesellschaft Für Naturforschung, Senckenberganlage 25, 60325 Frankfurt, Germany

## Correction to: BMC Ecol Evo (2019) 19:11 https://doi.org/10.1186/s12862-018-1324-9

Following publication of the original article [1], we were notified that the Dryad Digital Repository link under "Availability of data and materials" was missing a dot. The correct link is: https://datadryad.org/stash/dataset/doi:10.5061/dryad.55p667b.

The original article has been corrected.
