# Supplementary figures and images for: Diversity and evolution of the transposable element repertoire in arthropods with particular reference to insects
Source: BMC Evol Biol. 2019 Jan 9;19:11. doi: 10.1186/s12862-018-1324-9 (PMC6327564; doi:10.1186/s12862-018-1324-9)

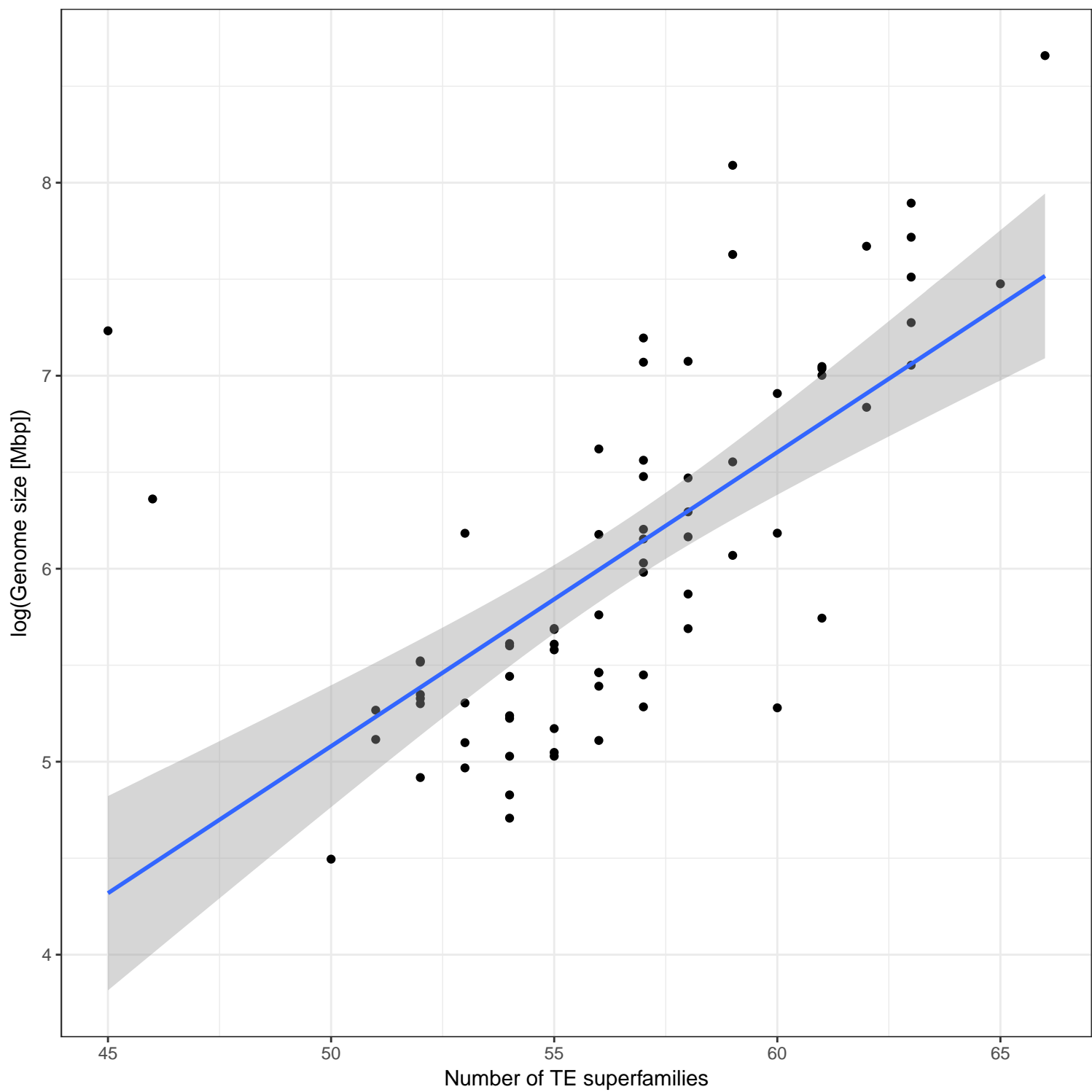

Supplement: Supplementary file 2 — This plot shows that the number of TE superfamilies is correlated to the genome assembly size. (PDF 7 kB) [file 12862_2018_1324_MOESM2_ESM.pdf]

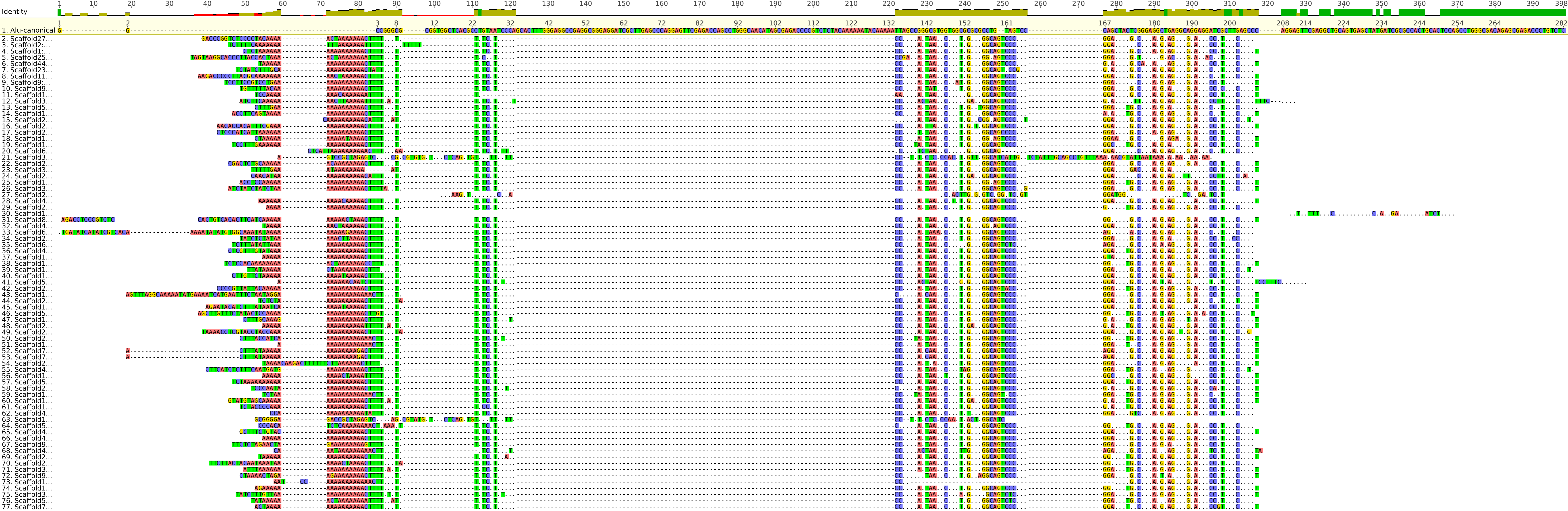

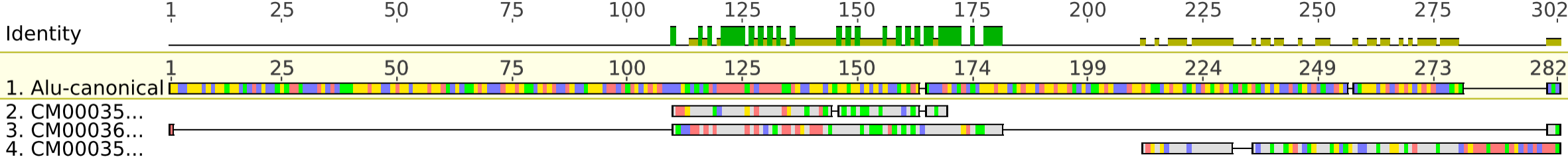

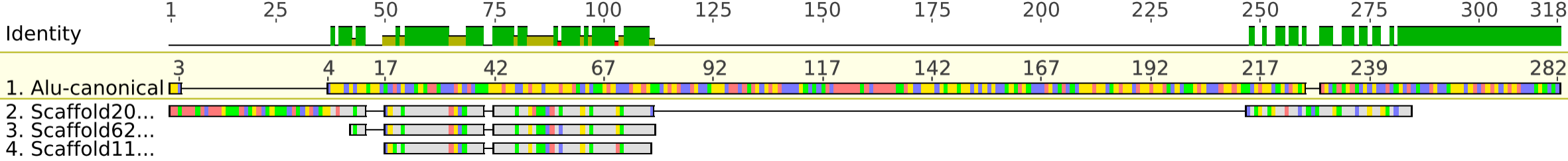

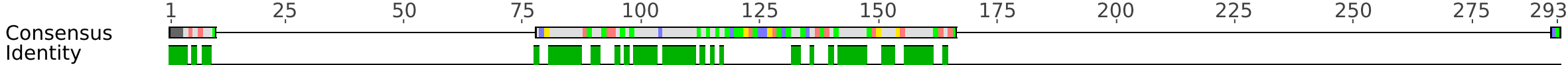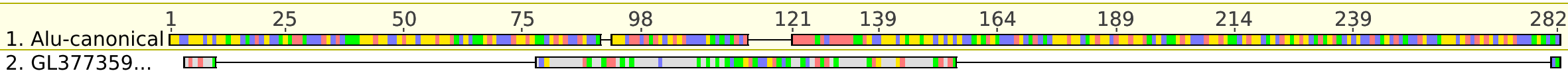

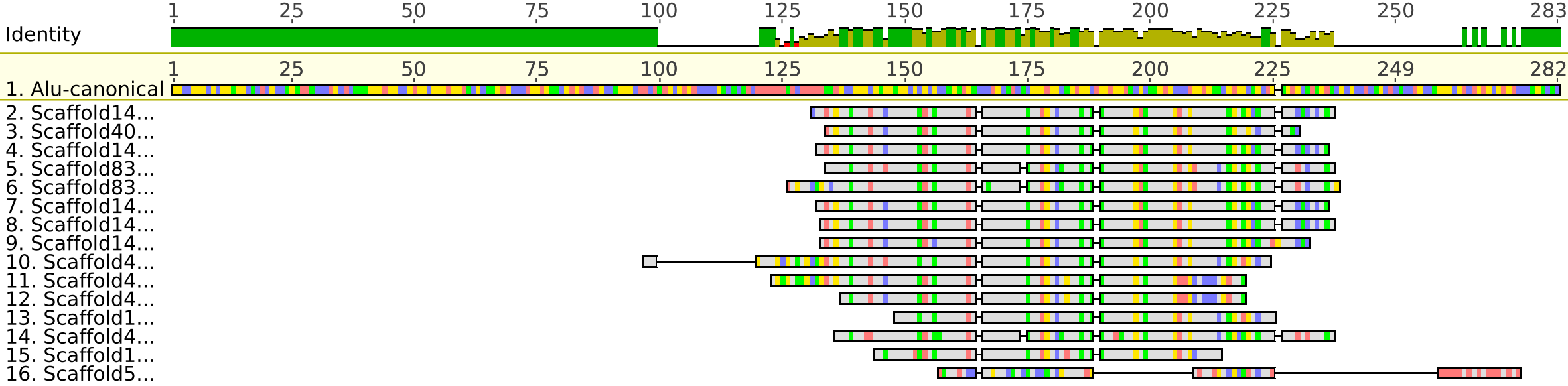

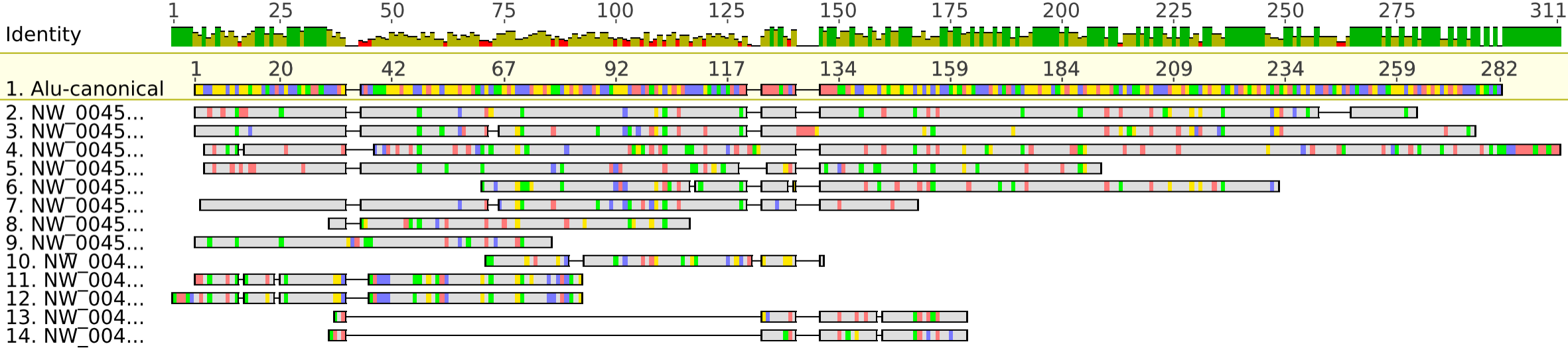

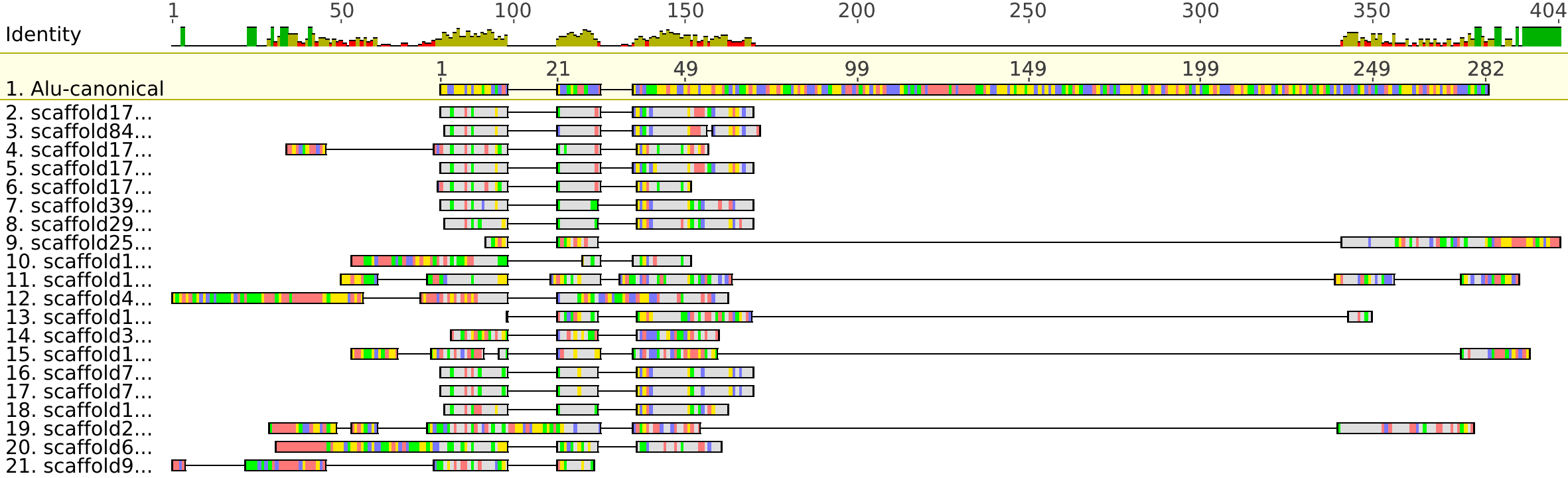

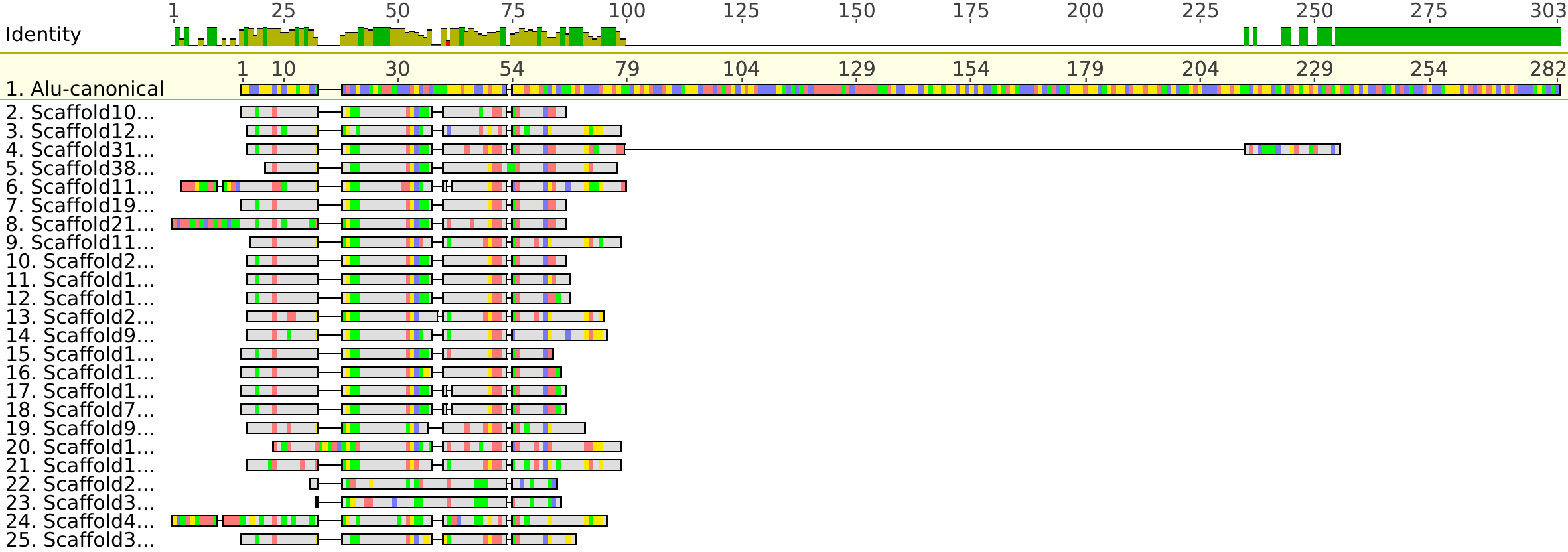

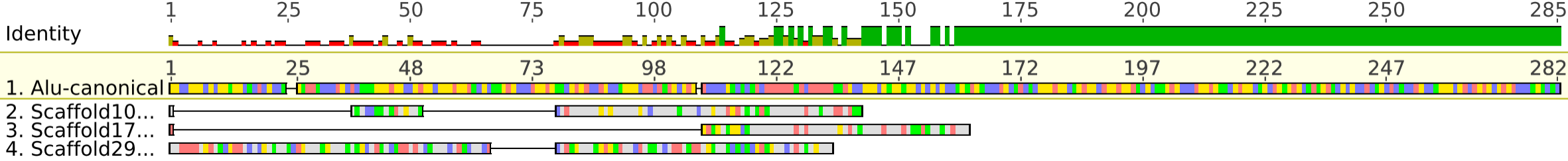

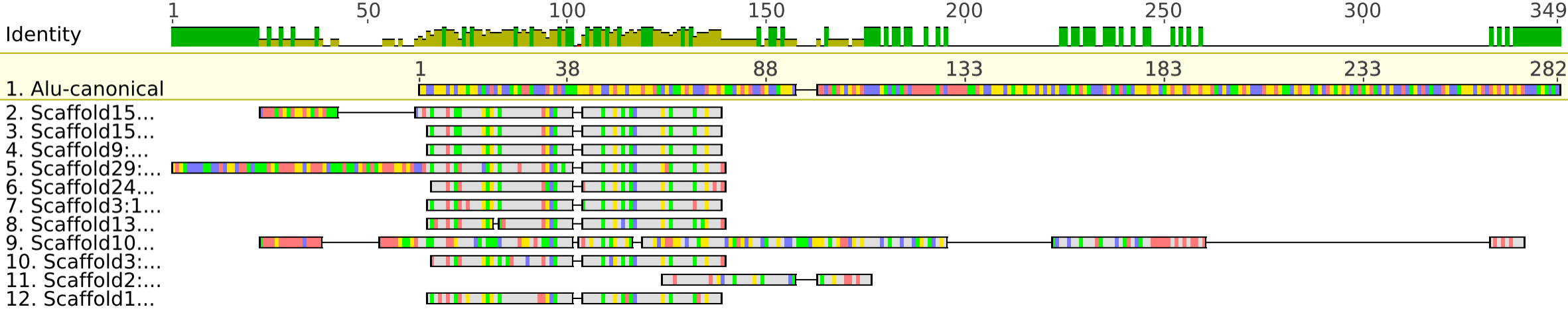

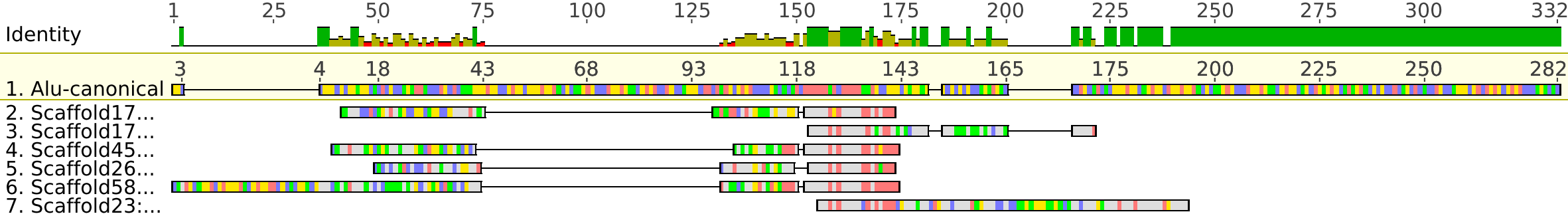

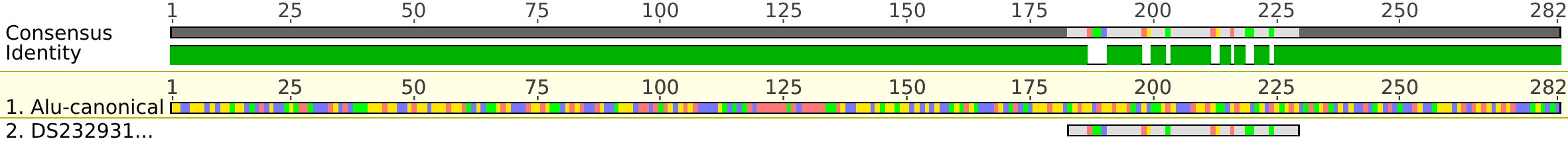

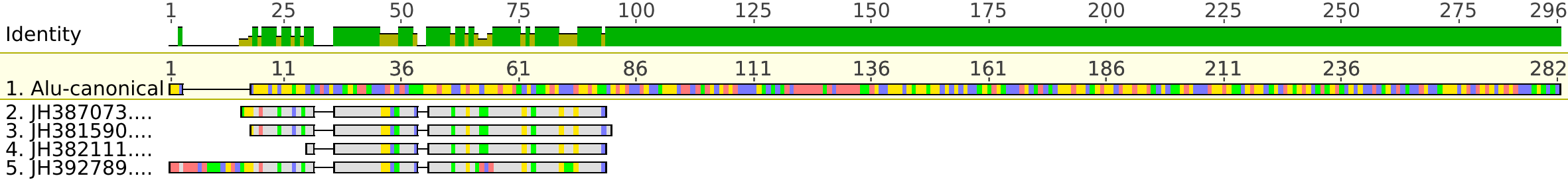

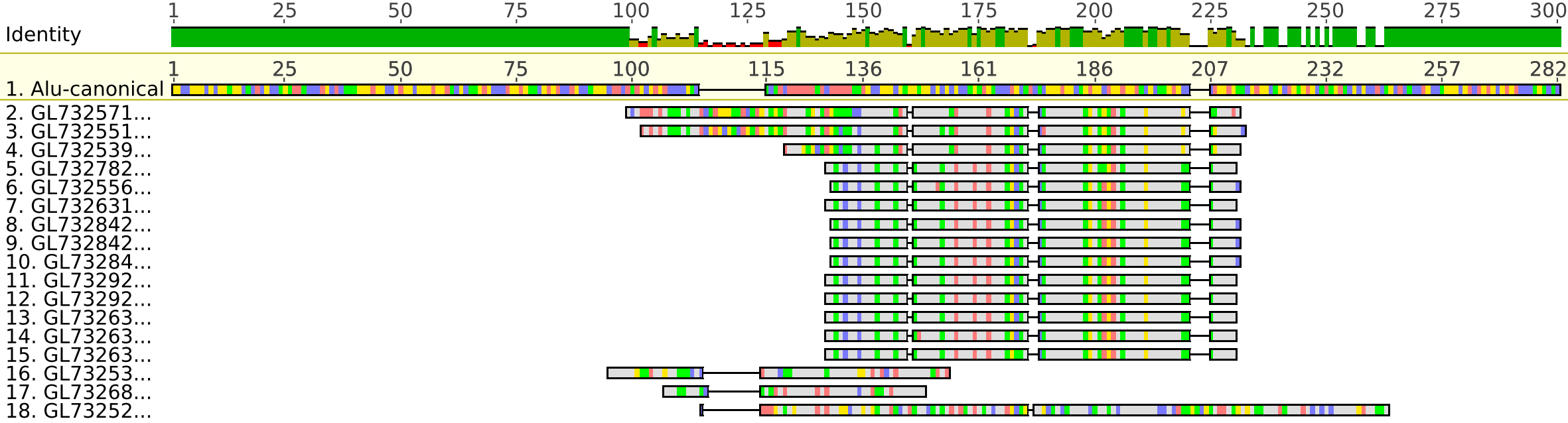

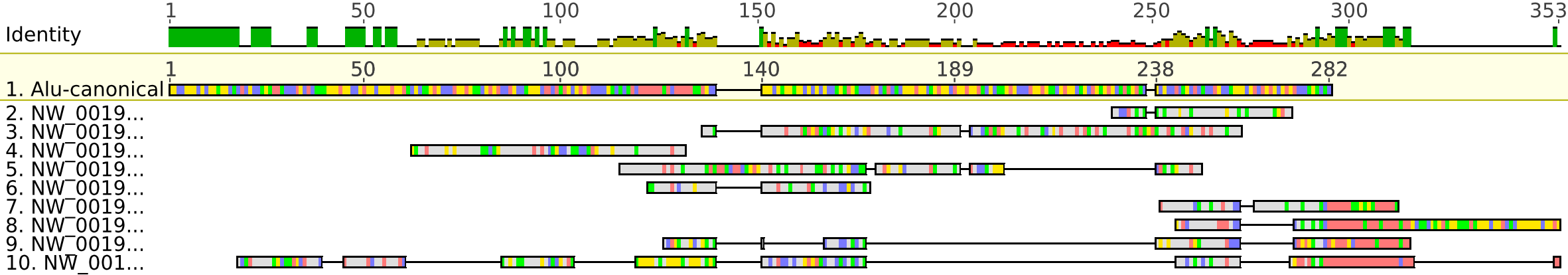

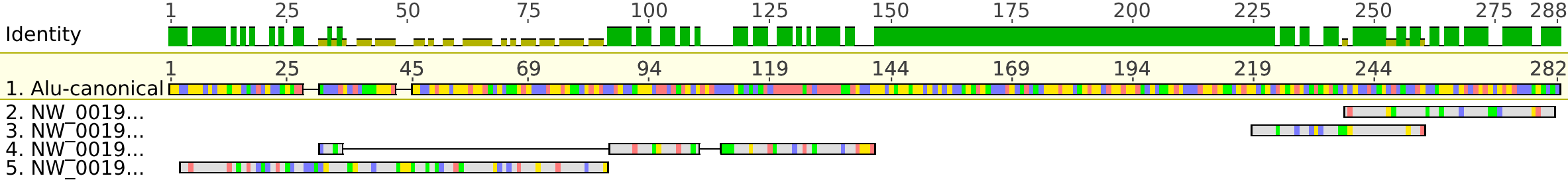

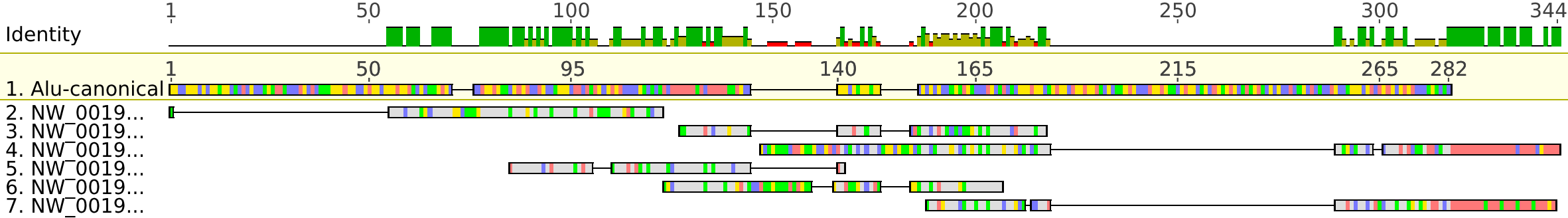

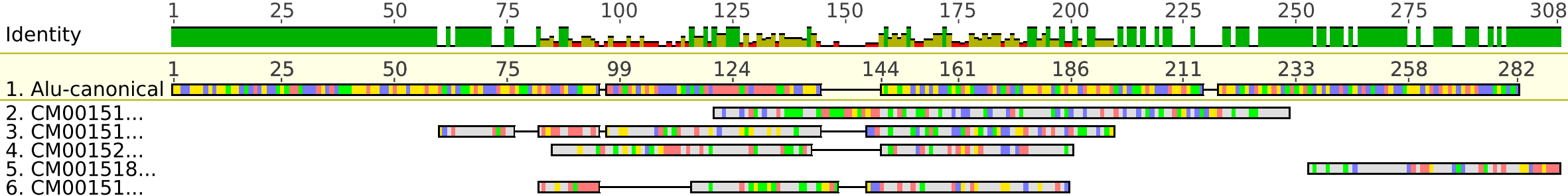

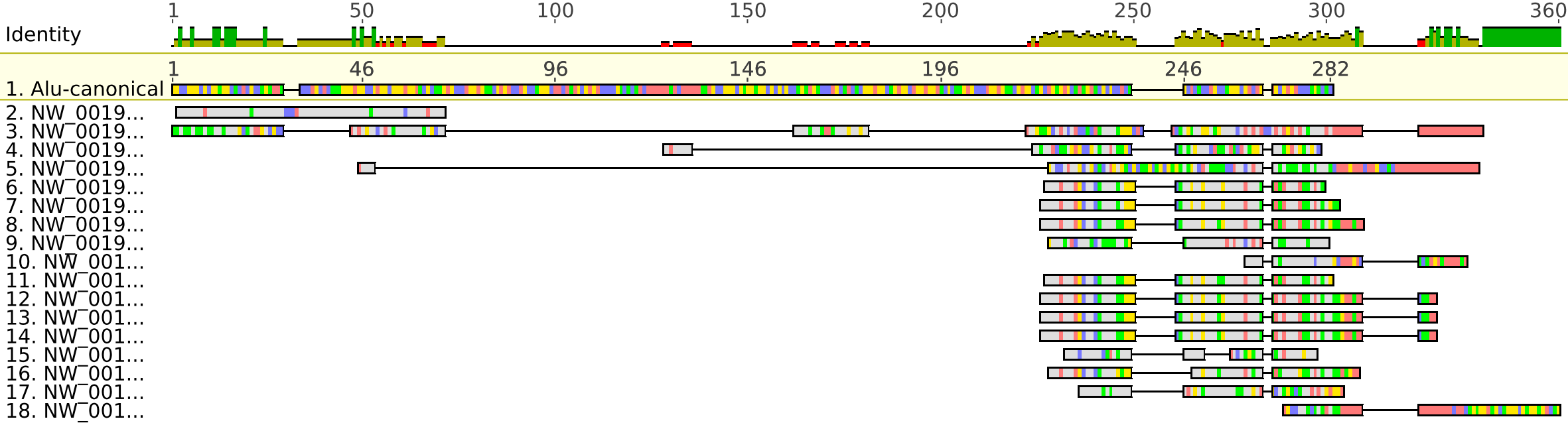

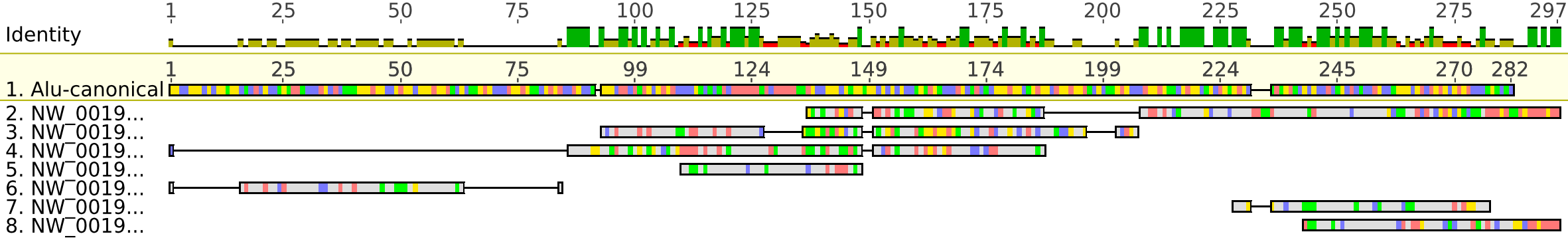

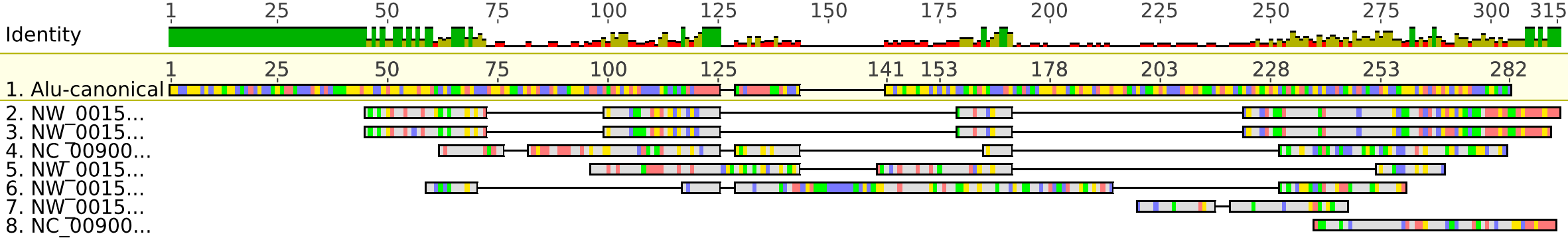

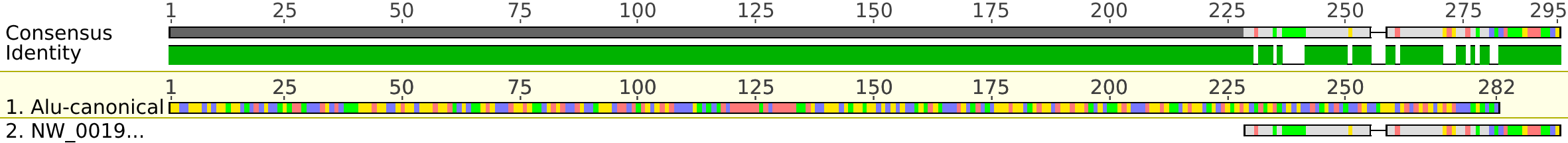

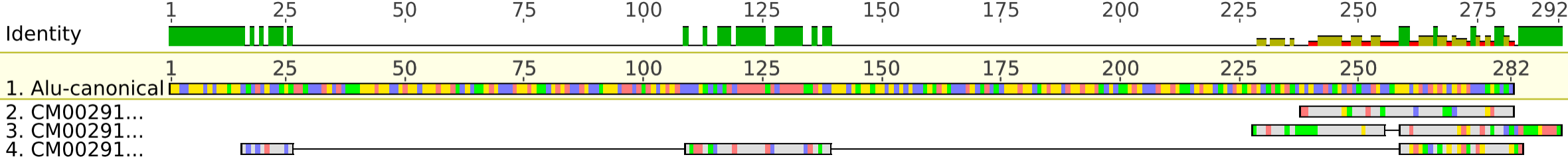

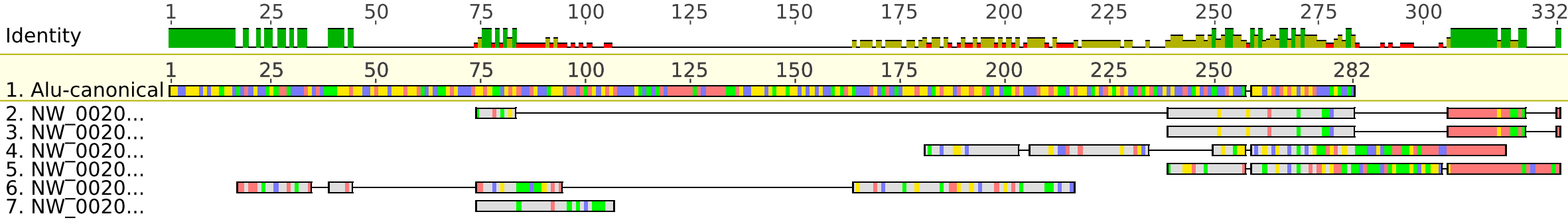

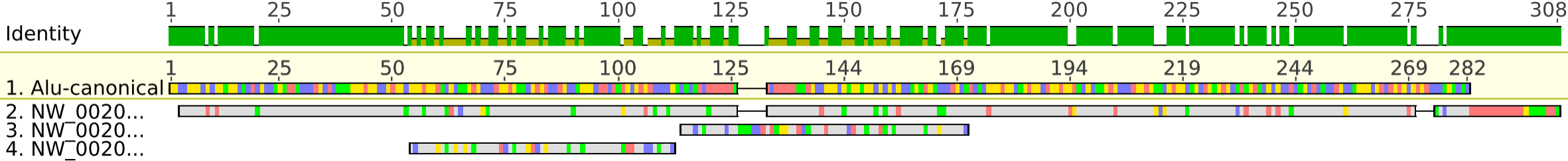

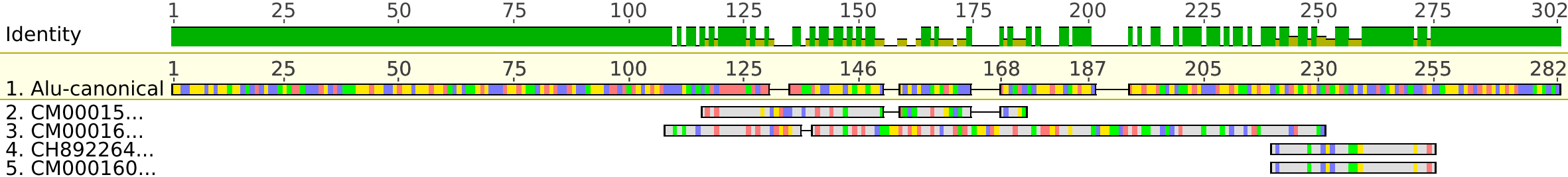

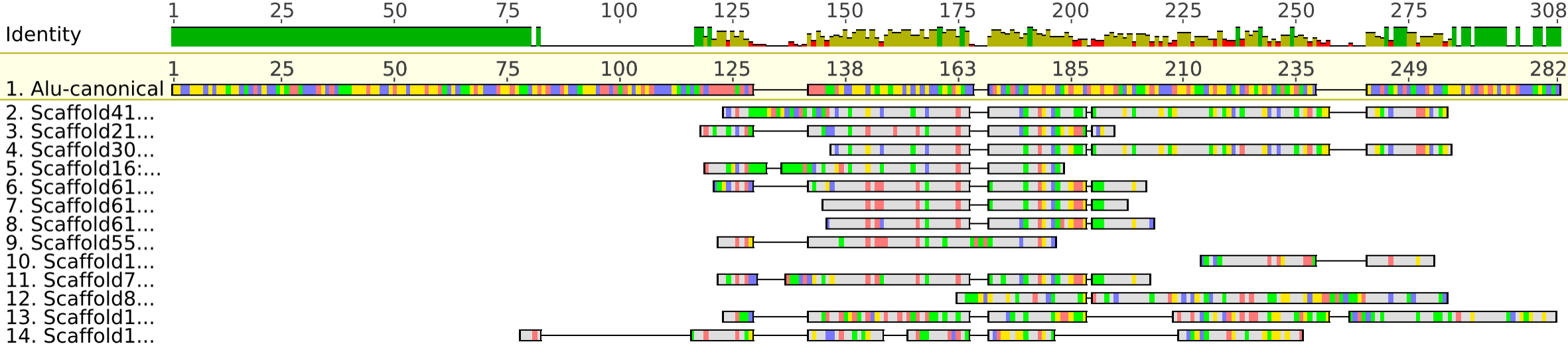

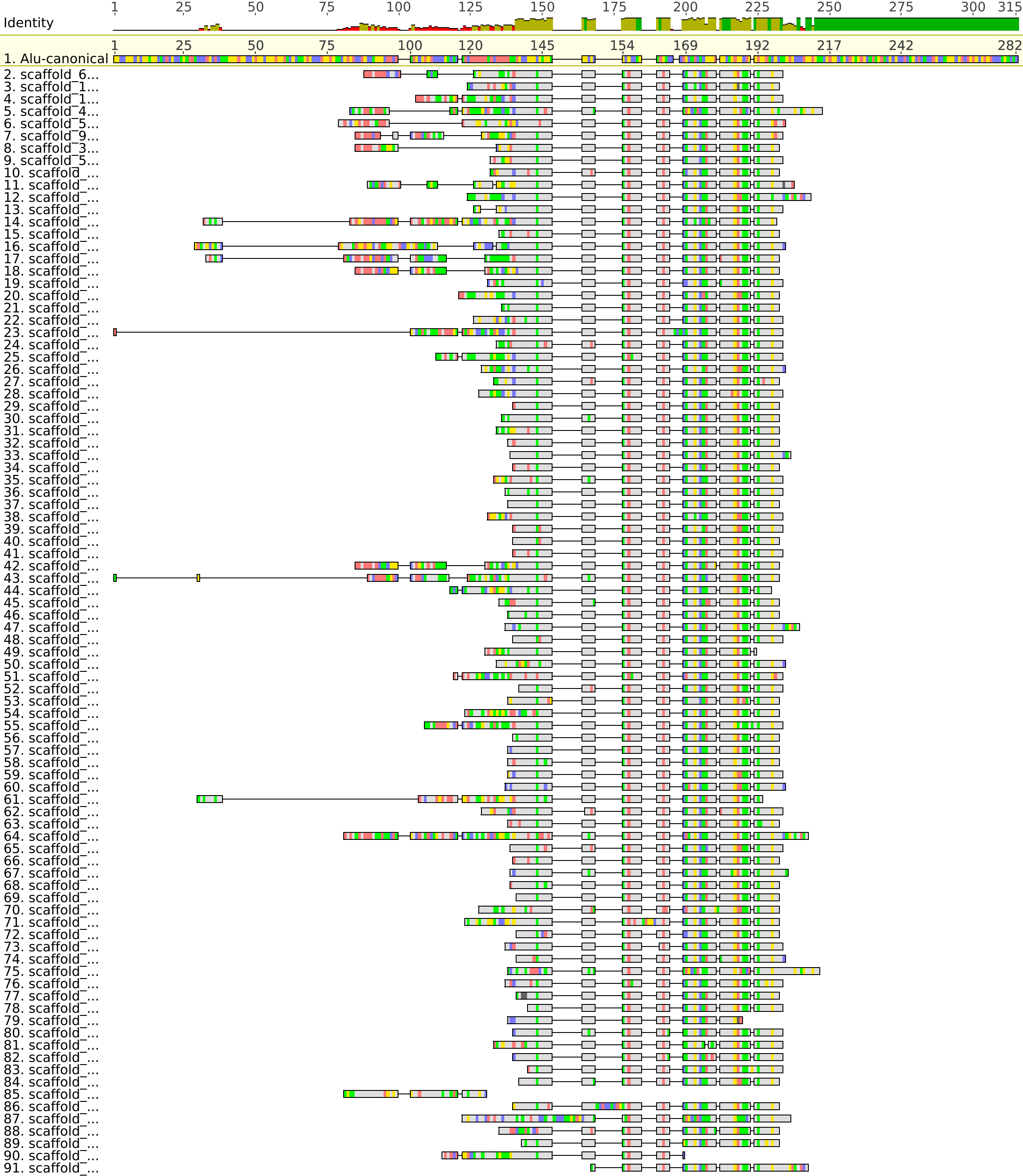

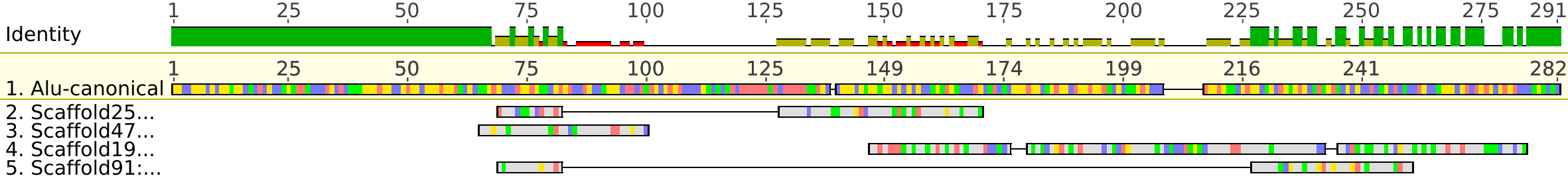

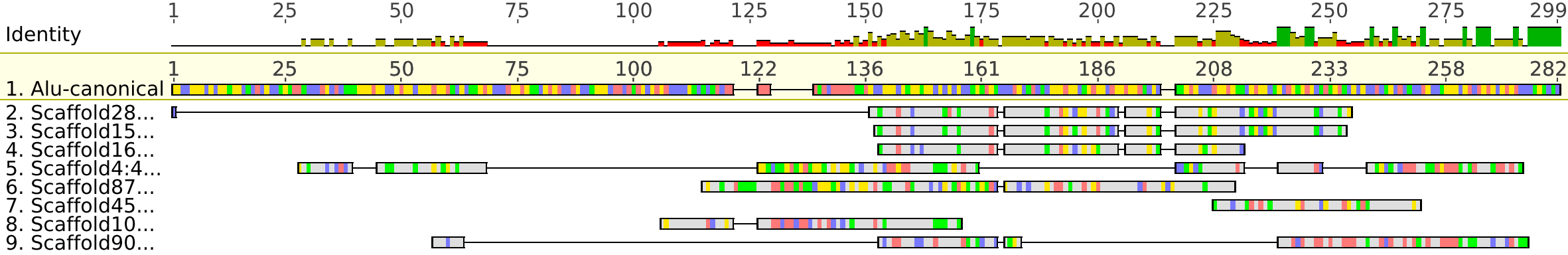

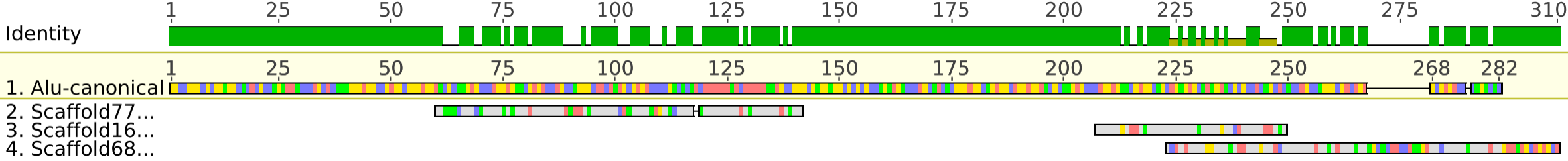

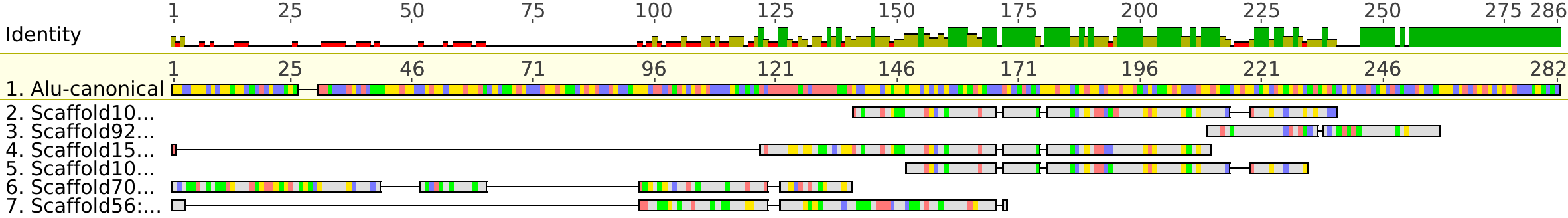

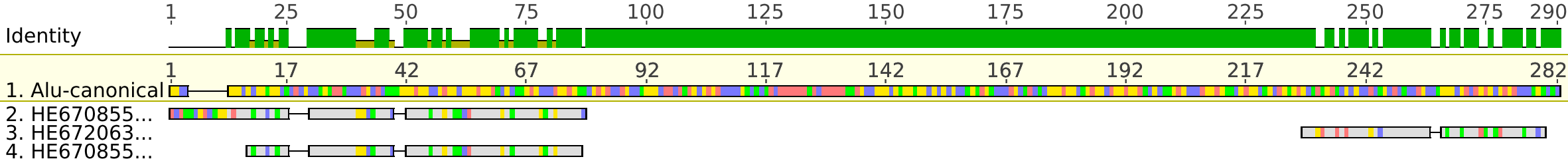

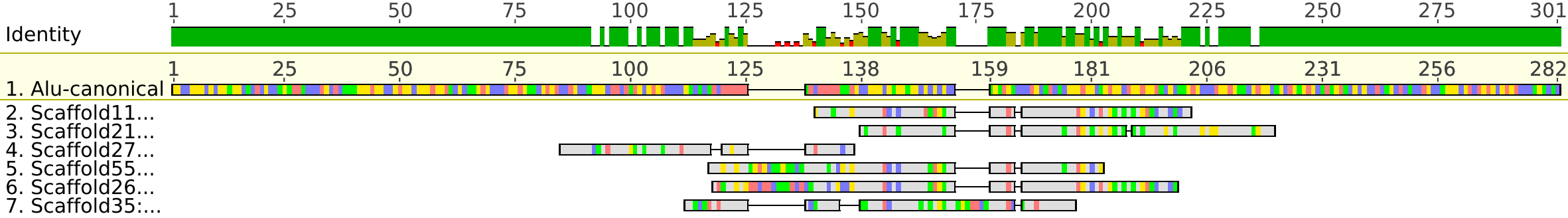

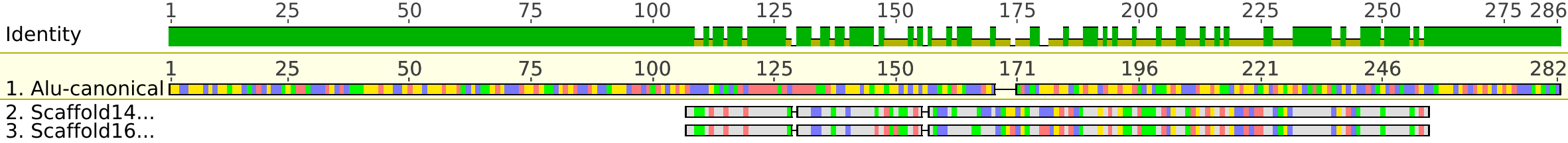

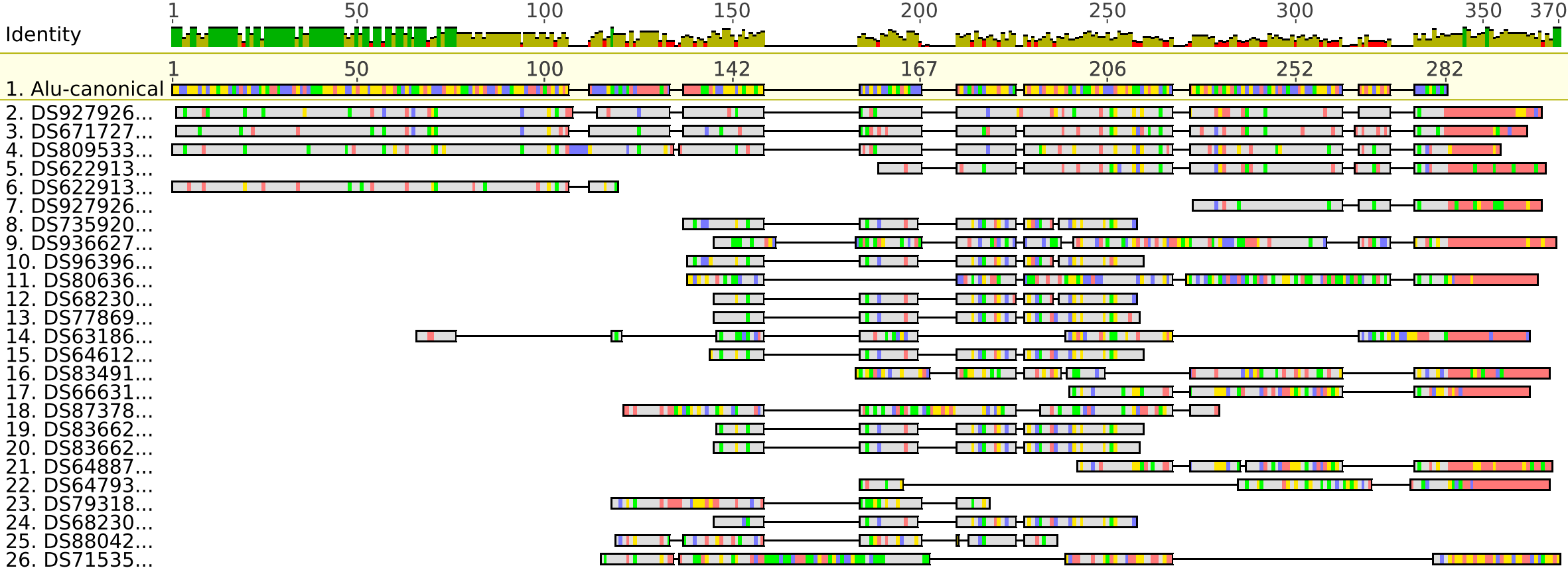

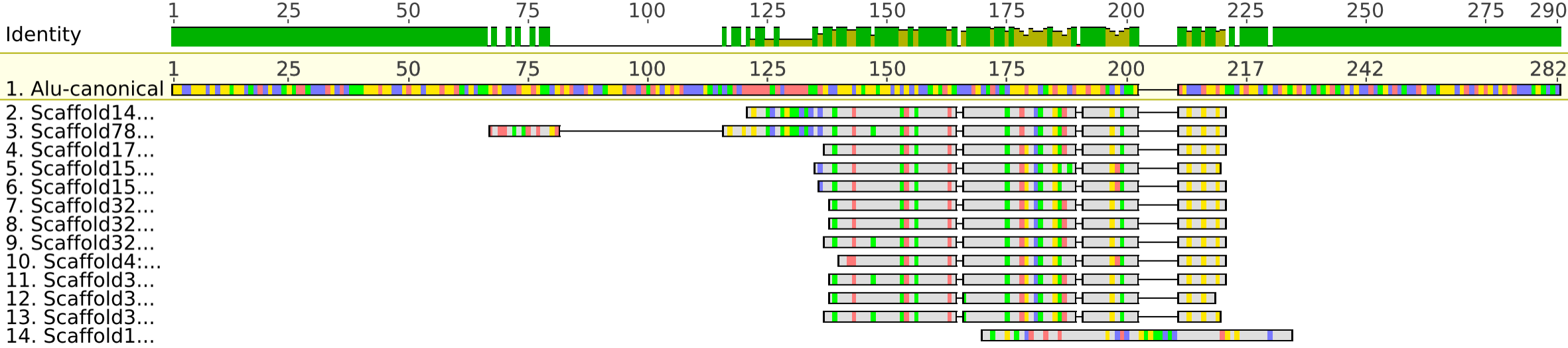

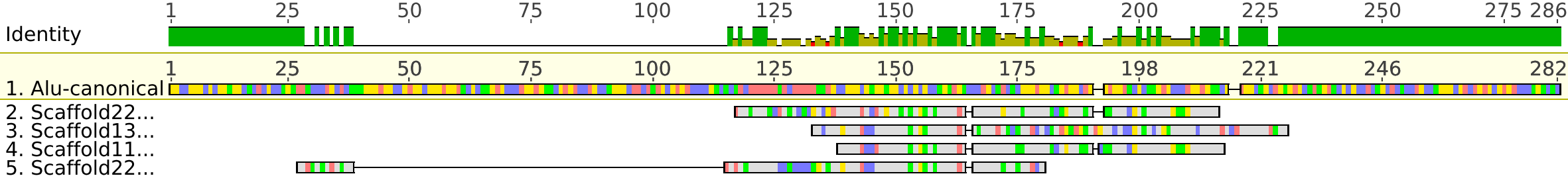

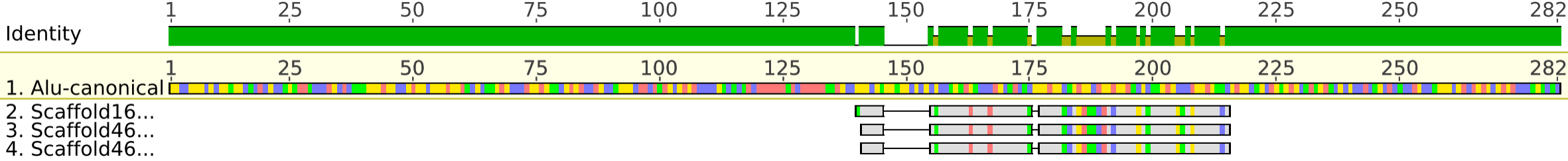

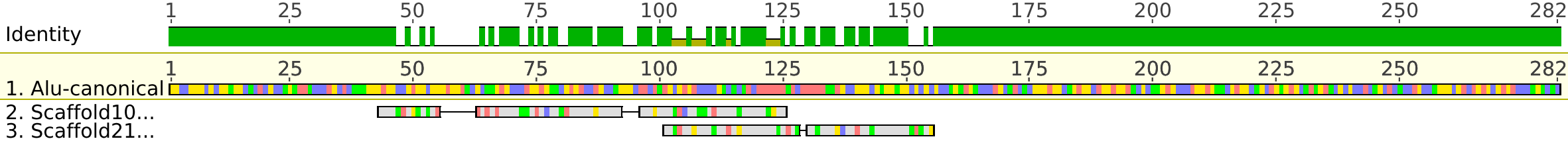

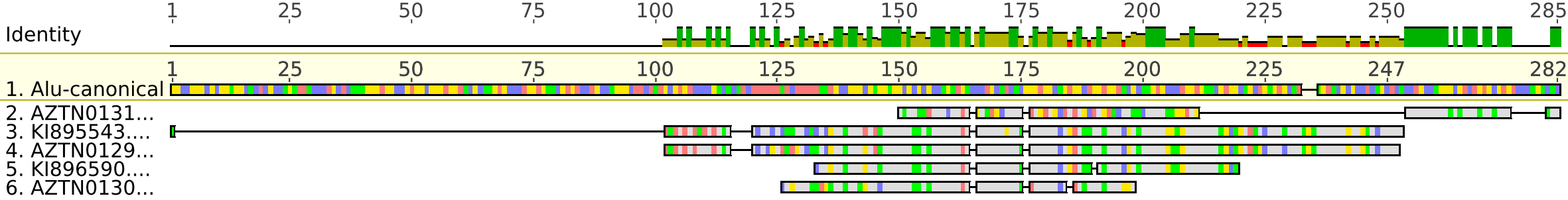

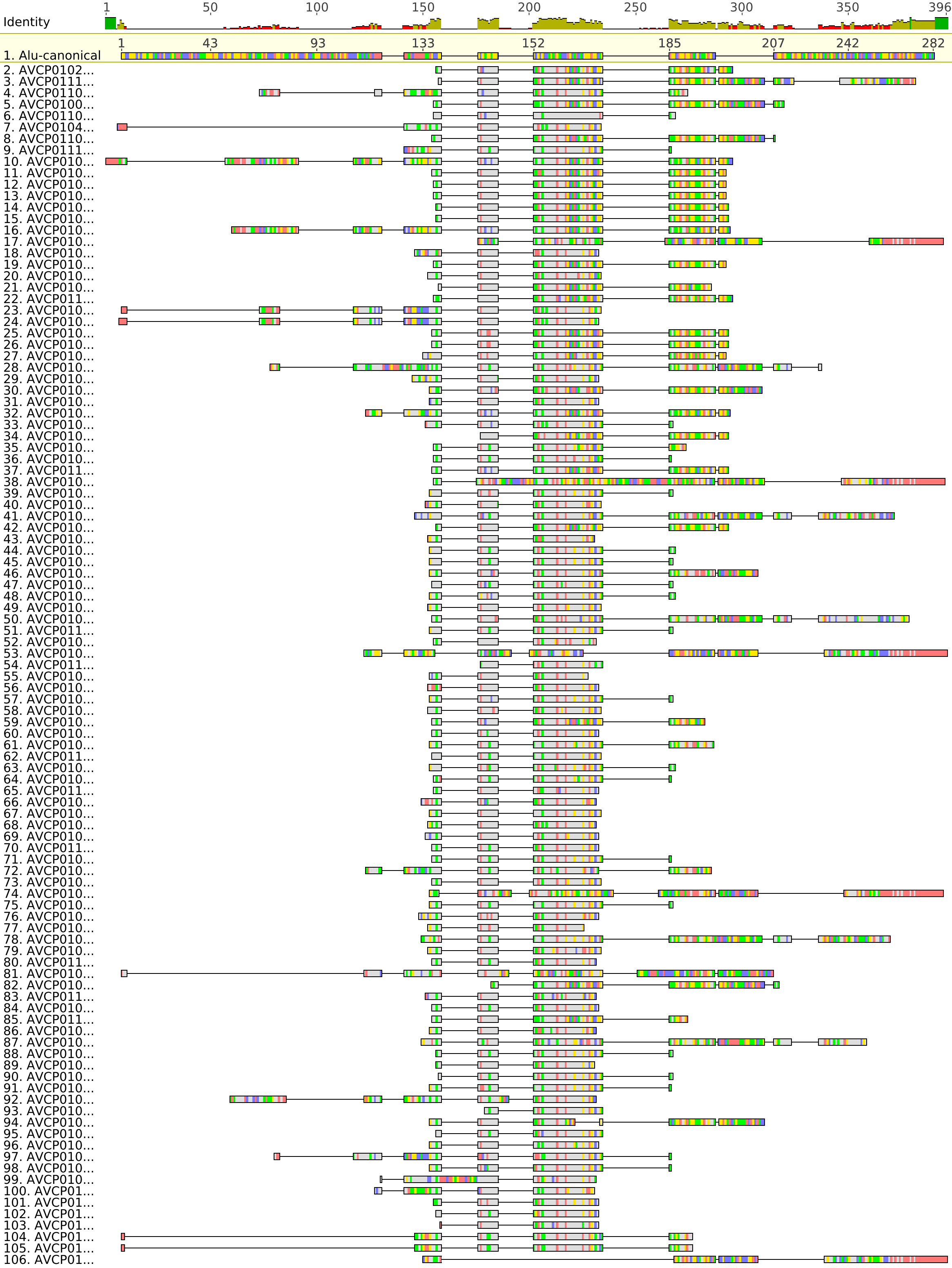

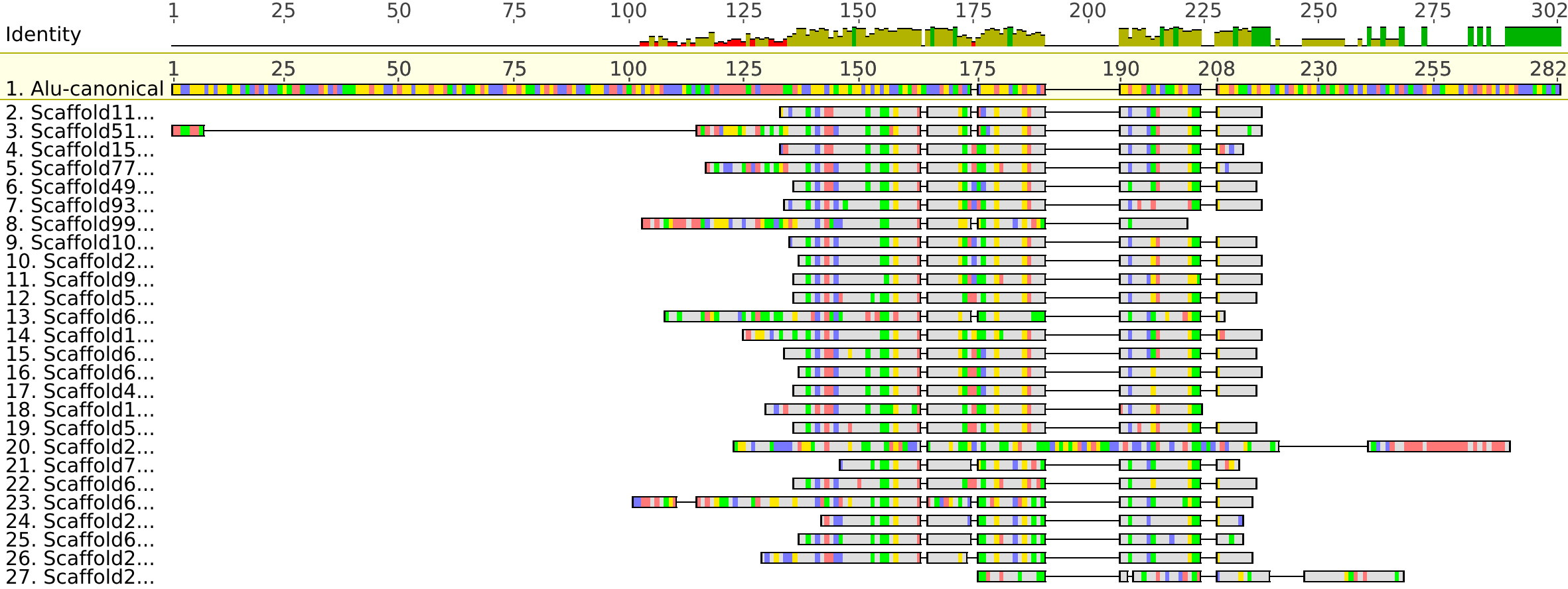

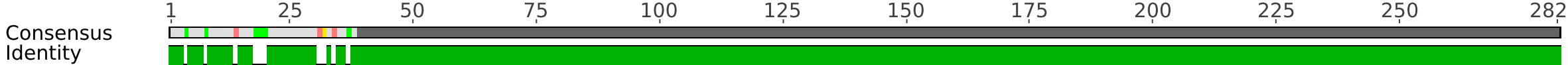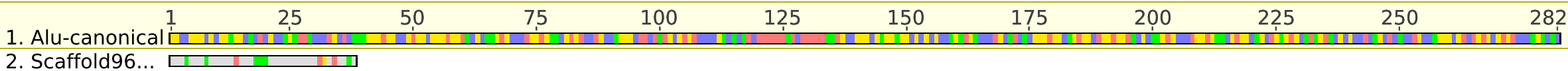

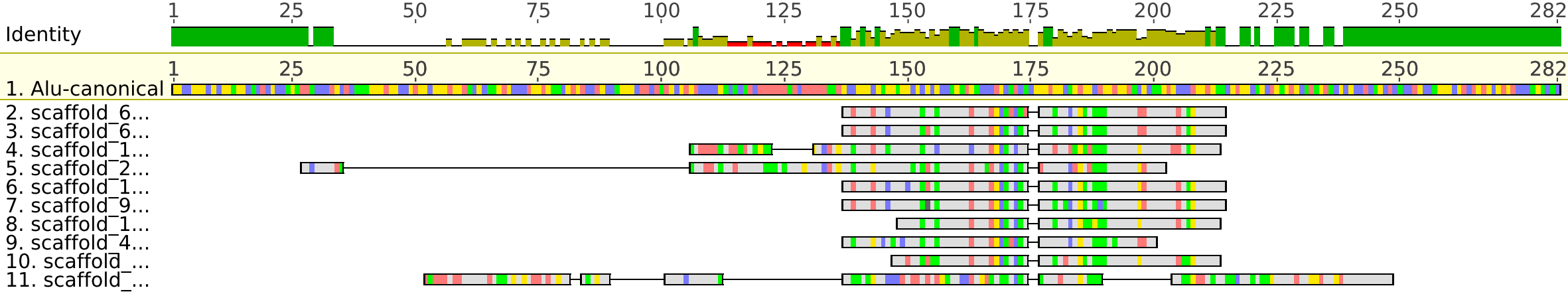

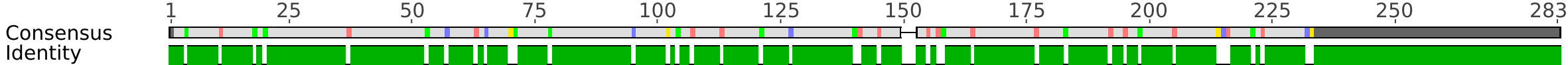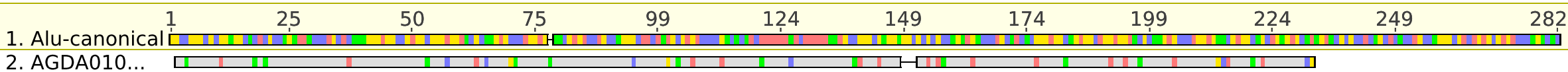

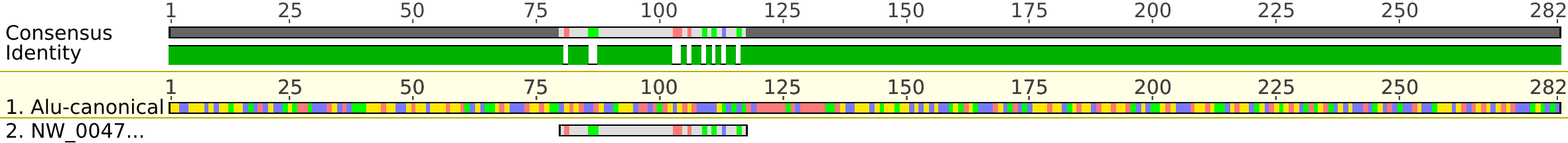

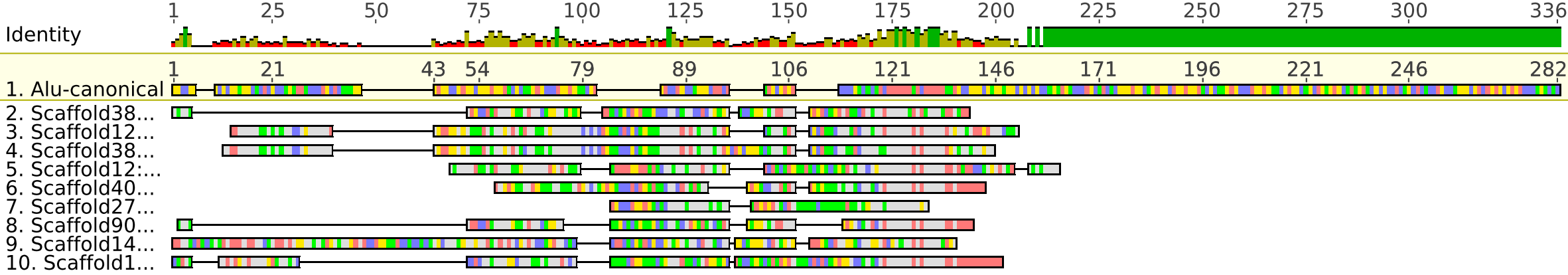

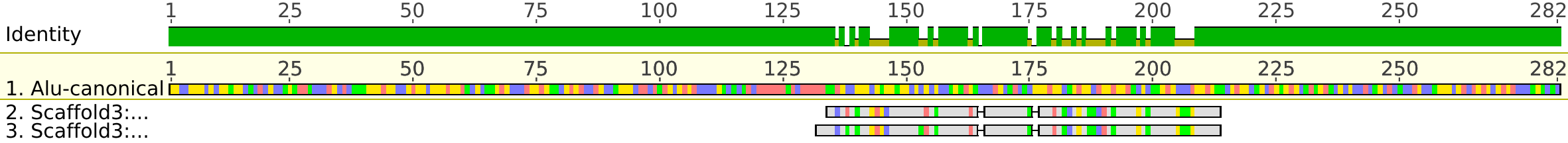

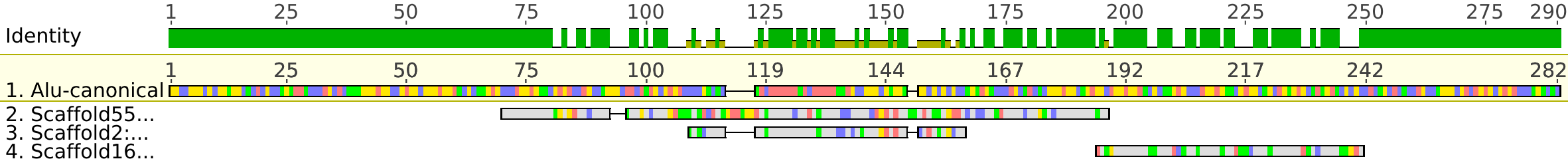

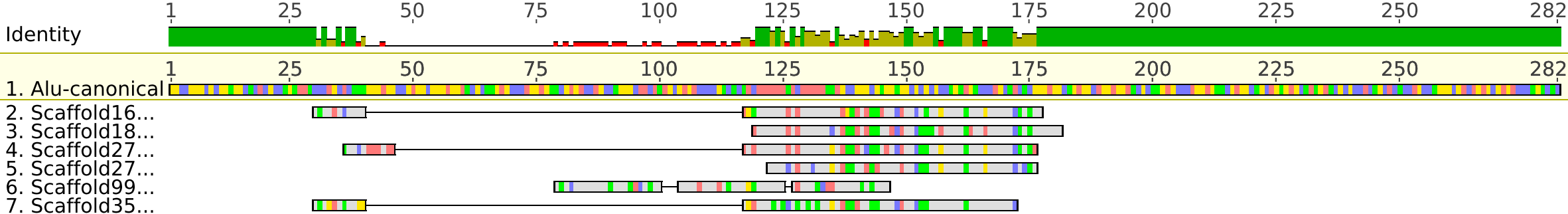

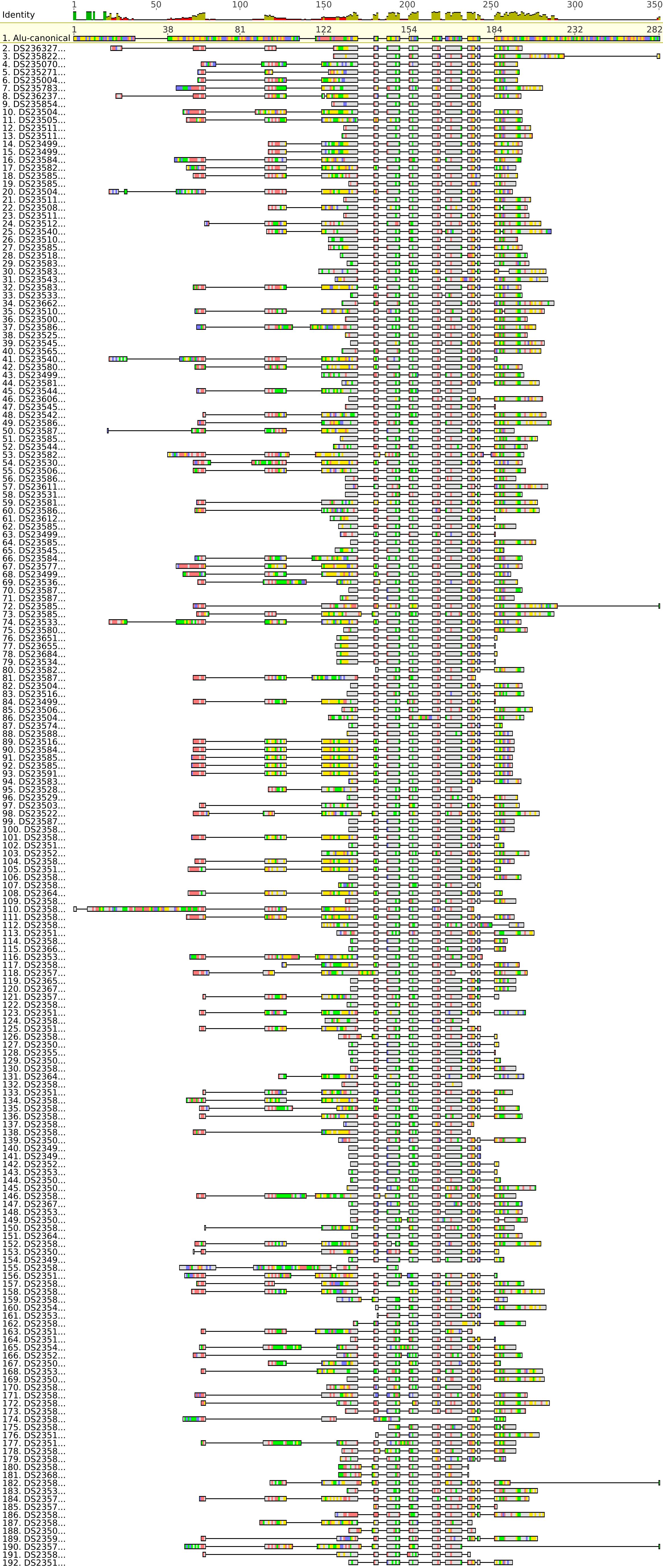

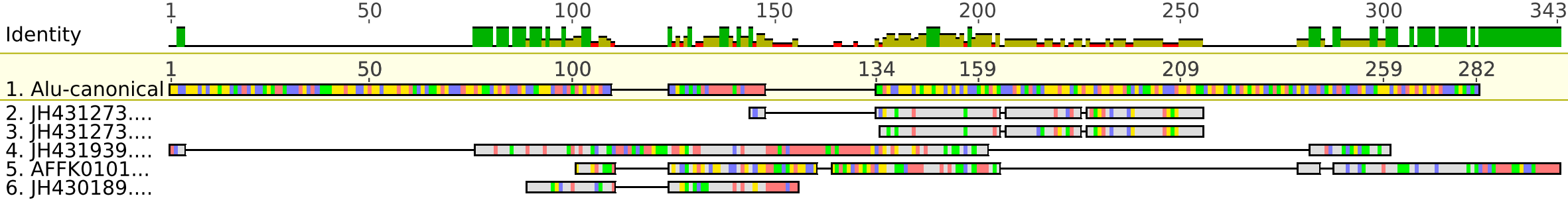

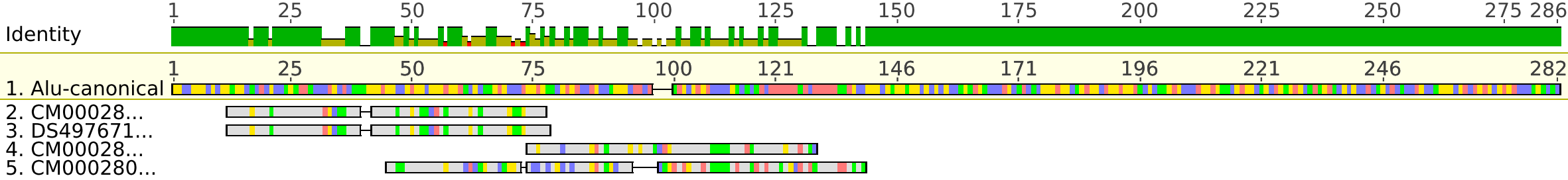

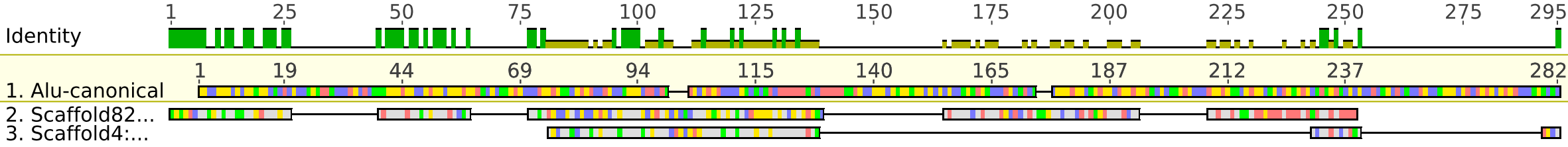

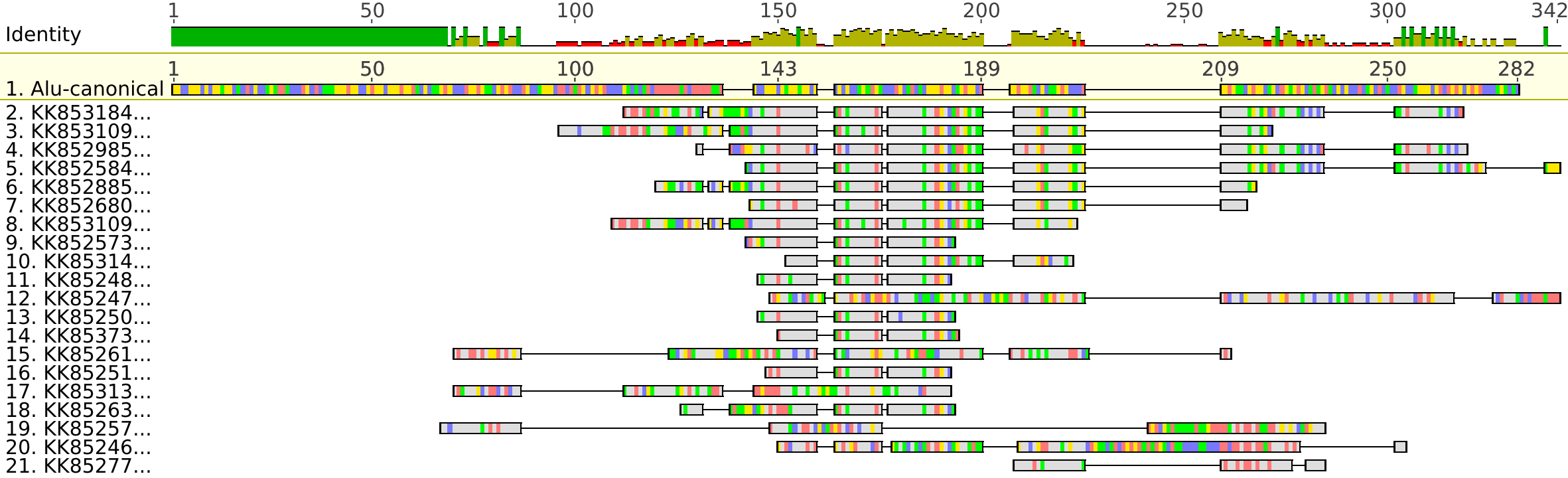

Supplement: Supplementary file 3 — Alu alignments. These plots illustrate that copies of the SINE Alu are present in 56 of the genomes under study. Grey sections in the alignments are positions identical to the canonical Alu sequence at the top. (PDF 4480 kb) [file 12862_2018_1324_MOESM3_ESM.pdf]
